# Plastic brain mechanisms supporting reduction in cravings induced by response training

**DOI:** 10.1162/IMAG.a.1180

**Published:** 2026-03-30

**Authors:** Malika Tapparel, Eva R. Pool, Hugo Najberg, Lucas Spierer

**Affiliations:** Laboratory for Neurorehabilitation Science, Medicine Section, Faculty of Science and Medicine, University of Fribourg, Fribourg, Switzerland; Department of Psychology, Faculty of Psychology and Educational Sciences, University of Geneva, Geneva, Switzerland; Swiss Center for Affective Sciences, University of Geneva, Geneva, Switzerland

**Keywords:** executive training, reward system, ERP, electrical neuroimaging, incentive saliency, Pavlovian learning

## Abstract

Recent evidence indicates that interventions involving the repeated inhibition of motor responses to environmental cues reduce brain reward responses and craving for these cues, and in turn their consumption. Such approaches have been put forward as a promising solution to unhealthy food overconsumption. Yet, the neurocognitive mechanisms underlying inhibition training to reduce reward responses remain largely unknown. We addressed those questions in a within-participant pre–post intervention study examining how target stimuli (Go)/non-target stimuli (NoGo) training reduces implicit motivational and electrophysiological activity to trained sugary drink cues. We then examined whether computational indices of Pavlovian and other reinforcement learning biases moderate the efficacy of the training, to test how inhibition training involves learning mechanisms. In addition to replicating evidence for a reduction in explicit cue valuation with Go/NoGo training, our results identified the N2 event-related potentials (ERP) component as modulated by the training, and individual Pavlovian biases as moderators of training efficacy. These findings point to inhibitory control and associative devaluation as the neurophysiological mechanisms supporting the effect of Go/NoGo training on cue valuation, and a contribution of Pavlovian learning in these processes.

## 1 Introduction

Unhealthy consumption behaviours account for most non-communicable diseases such as diabetes, cardiovascular diseases, or cancer, which cause 70% of deaths worldwide (WHO, n.d.). Critically, these behaviours persist even after informing individuals about their negative impact on health or after they experience unwanted consequences (Marteau et al., 2012). This is due to eating behaviour being almost always a “negotiation” of rational control and automatic processes which are triggered by environmental cues (Marteau et al., 2012; Strack & Deutsch, 2004). For instance, the availability and visibility of energy-dense palatable foods parallel the prevalence of overweight and obesity (Boswell & Kober, 2016). These food cues (over)activate the brain reward system, which in turn induces craving and overeating. Elevated frontostriatal activity and motivational response to food cues indeed predict their consumption (Lawrence et al., 2012; Nijs et al., 2010; Nolan-Poupart et al., 2013; Werthmann et al., 2014) and are associated with individuals’ weight status (Frankort et al., 2012; Pursey et al., 2014).

Restoring healthy neurocognitive reward responses to problematic environmental cues might thus constitute a powerful lever to reduce overconsumption. Such effects can be achieved by the repeated inhibition of motor responses to unwanted cues: the practice of executive control Go/NoGo (GNG) tasks, in which participants must respond to target stimuli (Go) and withhold their responses to non-target stimuli (NoGo), decreases their perceived value and consumption (Allom et al., 2016; Jones et al., 2016; Stice et al., 2016; Veling et al., 2017). A prevailing hypothesis about the underlying mechanisms of GNG training is the Behaviour Stimulus Interaction theory (Chen et al., 2016; Veling et al., 2008), which posits that appetitive stimuli elicit strong approach responses that are devalued following the active suppression of those responses in GNG training (see section 2.6). Another hypothesis involves the formation of automatic associations between certain food items and the act of stopping, potentially involving the activation of a “stop centre” in the brain or rapid suppression of motor cortex excitability (Best et al., 2016; Veling et al., 2017; Verbruggen et al., 2014). Over time, this requirement for inhibitory control decreases as direct stimulus-stop associations are acquired, leading to an automatic inhibition response. Lastly, a less emphasised hypothesis (Veling et al., 2017, 2022) stipulates that this devaluation results from the training of top–down “inhibition reflexes” towards trained stimuli (Manuel et al., 2010; Spierer et al., 2013; Veling et al., 2011; Verbruggen et al., 2014).

The associative learning mechanism putatively supporting the effects of food response training, together with observations that these interventions reduce the motivational saliency of the target items, suggest that learning mechanisms such as Pavlovian and other reinforcement learning biases might be involved in the effect of food response training. Pavlovian learning, also known as classical conditioning, involves the acquisition of an affective value by a neutral stimulus (conditional stimulus, CS) as it predicts a rewarding outcome (unconditional stimulus, US), and individuals show differences in this affective value’s learning process (Flagel et al., 2011; Wuensch et al., 2021). In sign-tracking, individuals upon observing a conditional stimulus signaling an impending reward are prone to approach and interact with that signal itself, treating the predictive signal as the desired outcome. Whereas in goal-tracking, individuals exposed to the same conditioned stimulus tend to rely on a different representational structure focusing on the association between the conditioned stimulus and a representation of the reward rather than the signal; the behaviour is thus specific to properties of the unconditioned stimulus. This learning bias can be measured through a classical Pavlovian conditioning paradigm paired with eye-tracking in which sign-tracking is expressed by a higher fixation time on the CS position versus on the US position for goal-tracking (Garofalo & di Pellegrino, 2015; Schad et al., 2020). In reinforcement learning tasks involving action and choices, a value is attributed to an individual’s action by updating the expected value of this action based on their precedent outcome (Sutton & Barto, 2018). In this learning process, the model-free system learns an action’s value solely through reward prediction errors with minimum planning and adjustment to the current goal, whereas the model-based system integrates a more complex strategy, building an internal model of the environment to predict future states and outcomes (Daw et al., 2005; Dayan & Niv, 2008). The relative contribution of those learning mechanisms in the decision-making process can be measured with a two-step task, in which participants initially choose between stimuli that probabilistically lead to different second-stage options, each offering further choices linked to varying chances of reward (Daw et al., 2011). An individual’s bias lies in their balance between sign-tracking/model-free learning or goal-tracking/model-based learning (Flagel et al., 2011). Thus, while sign-tracking/model-free learning is oriented towards the predictive stimulus and promotes a habitual response, goal-tracking/model-based focuses on the predicted outcome. Accordingly, we posit that individuals biased towards sign-tracking/model-free learning will be more sensitive to reward-associated cues (Flagel et al., 2011) and will thus pay more attention to the cues prompting response inhibition. Hence, they will show larger responses to GNG interventions (Flagel et al., 2011).

However, these hypotheses lack empirical support, preventing the development of neurophysiologically and individually optimised interventions (Aulbach et al., 2020; Carbine et al., 2021; Stice et al., 2017; van de Vijver et al., 2018; Yang et al., 2023). Elucidating the neural correlates of executive control training is indeed necessary to improve the efficacy of these behavioural change interventions through mechanism-based population stratification and to develop new interventions (Stice et al., 2017; Yang et al., 2023). Whereas previous studies have shown that response training effectively reduces the explicit valuation of targeted items (Najberg, Rigamonti, et al., 2021; Najberg et al., 2023), a gap remains about the effect of those interventions on the implicit motivational value of items. This question is crucial when evaluating response training’s effect because explicit measures such as the “explicit liking” self-reported likeability rating, lack consistency, and might be confounded (Pool et al., 2016). Likewise, bridging the gaps between the learning and executive training literature will pave the way for the identification of new prognostic markers of overconsumption-related conditions (Garofalo & di Pellegrino, 2015; Schad et al., 2020). The overvaluations of environmental cues at the origin of these conditions indeed likely proceed from the same associative learning mechanism as those inducing item devaluation during response training. Current personalisation strategies in food response training target preferred food items but do not focus on individuals’ traits (Manasse et al., 2020; Najberg, Rigamonti, et al., 2021; Najberg et al., 2023). Understanding the contribution of learning bias in response training would constitute an important step towards finer tailoring of interventions, which would, in turn, improve their efficacy.

To address these questions, we conducted a within-participant pre–post intervention study focusing on the effects of food GNG training tailored to participants’ preferences on the behavioural and electrophysiological responses to the trained cues. We compared the effect of training on preference-matched sugary drink items randomly assigned to a “Go” or “NoGo” condition to isolate the intervention’s mechanisms of action while controlling for item exposure and participants’ expectations in a fully within-subjects design.

We then combined this approach with computational analyses of Pavlovian and other reinforcement learning biases to identify whether learning endophenotypes moderate the efficacy of response training. We used the gaze direction to index Pavlovian bias by computing the fixation time on the conditioned minus on the unconditioned stimulus regressed on the true conditioned stimulus value (as in Schad et al., 2020). To evaluate other reinforcement learning biases, we computed the relative contribution of model-free and model-based systems in the net value of the action and extracted the weighting parameter *w* (according to the procedure of Daw et al., 2011; Voon et al., 2015).

Each research question and related hypothesis is given in Table 1. The full design table is given in the Supplementary Material (Supplementary Table S2).

**Table 1.** Hypotheses.

Research question	Tested hypothesis
Will the intervention decrease the explicit liking of “NoGo” trained items?	**HR:** We expect a larger pre- post-training reduction of the explicit liking in the “NoGo” than “Go” items.
Will we observe neural correlates supporting the hypothesised mechanisms of action of food response training?	We expect different pre- to post-training modulations of the GFP and GMD indexes during the correct withholding responses of items trained as “Go” and trained as “NoGo” during the intervention. **H1a:** These differences should be expressed in the P1 ERP occipital component at 50–150 ms post-stimuli onset, associated with attentional saliency. No direction in these modulations is expected. **H1b:** These differences should be expressed in the N2 ERP frontocentral component at 150–300 ms post-stimuli onset, associated with motoric inhibition. No direction in these modulations is expected.
Will the intervention decrease the implicit wanting in “NoGo” trained items?	**H2:** We expect a larger decrease in the “NoGo” than in the “Go” items’ implicit wanting between pre- and post-training.
Will learning bias moderate the intervention’s efficacy?	**H3a:** We expect that higher sign-tracking bias (higher regression coefficient) will be associated with a larger reduction in the explicit liking (delta between pre- and post-training) of trained as “NoGo” items. **H3b:** We expect that a higher model-free bias (lower w) will be associated with a larger reduction in the explicit liking (delta between pre- and post-training) of trained as “NoGo” items.

We first replicated our previous findings for a larger decrease in the explicit valuation of trained as “NoGo” than trained as “Go” cues (Najberg, Rigamonti, et al., 2021; Najberg et al., 2023; Replicatory Hypothesis, HR; Table 1; Supplementary Table S2).

At the neurophysiological level, we identified the mechanisms of food response training based on the pattern of electrophysiological change it induced, as assessed by the interaction term of a within-subject Item Category (“Go”, “NoGo”) by Session (Pre-, Post-training) design (H1; Table 1; Supplementary Table S2). If the food response training induces a reduction of cue valuation, it was expected to manifest as a modulation of the P1 event-related potentials (ERP) occipital component at 50–150 ms post-stimuli onset, an occipital processing step indexing attentional saliency (H1a) (Dickinson & Balleine, 2002; Ferrey et al., 2012; Houben et al., 2012; McLaren & Verbruggen, 2016; Schacht et al., 2012; Veling et al., 2008; Wiers et al., 2011; Zahedi et al., 2020). If the response training develops an “inhibition reflex”, it was expected to manifest as a modulation of the N2 frontocentral component at 150–300 ms associated with the detection and resolution of the conflict between response tendency driven by the orbitofrontal and ventromedial PFC reward activity and the need for inhibition (H1b) (De Pretto et al., 2019). To identify the neurophysiological plastic mechanisms supporting these mechanisms of action, we analysed two global features of the scalp field potential: its power and topography. This approach enables us to distinguish between strength- vs network-based plastic mechanisms: a change in global field power (GFP) without concomitant topographic modulation indeed indicates purely quantitative variations in the response of identical configurations of intracranial generators (Michel & Murray, 2012). In contrast, since topographic modulations necessarily follow from a change in the configuration of the underlying generators (Lehmann & Skrandies, 1980), a change in global map dissimilarity indices (GMD; with or without GFP modulations) indicates qualitative modulations of the network generating the ERPs. Because improved inhibitory performance has been both associated with increases and decreases of GFP (Spierer et al., 2013), and may also manifest as qualitative changes (GMD), we did not expect a direction for H1.

At the behavioural level, we expected a larger decrease in the motivational value of the target in the trained as “NoGo” than trained as “Go” items (H2; Table 1; Supplementary Table S2). We assessed the implicit motivation for specific items by measuring their “implicit wanting” component with a Stimulus–Response Compatibility (SRC) task (Mogg et al., 2003; Tibboel et al., 2015), which indexes the incentive value of stimuli as a slowing down of (incongruent) avoidance motor responses to the wanted cues.

To test the hypothesis that the mechanisms of response training interventions overlap with those of learning, we examined whether individual learning biases, as measured with a Pavlovian conditioning task with eye-tracking and a sequential learning task, moderate the effects of the intervention on its most replicated index of cue explicit liking (H3; Table 1; Supplementary Table S2).

## 2 Materials and Methods

### 2.1 Ethics information

All procedures were approved by the *Commission cantonale (VD) d’éthique de la recherche sur l’être humain* (CER-VD; #2024-00118) and performed in accordance with the relevant guidelines and regulations, as well as the Declaration of Helsinki.

### 2.2 Sampling plan

For all analyses, we determined their respective smallest effect size of interest (SESOI) on principled grounds. Even if a smaller effect than the SESOI could be expected, it was not considered of interest from an applied perspective (Anvari et al., 2021).

#### 2.2.1 HR) Training-induced modifications of explicit liking of the “NoGo” trained cues

We determined the SESOI for the explicit liking analyses to be a Cohen’s partial f of .25 (medium effect size, see also our two previous registered reports with this dependent variable; Najberg, Rigamonti, et al., 2021; Najberg et al., 2023). A smaller effect would not be interpreted as positive even if they reach our significance threshold. A-priori power analysis conducted with G-Power (Faul et al., 2007) for a 2 × 2 within-subject ANOVA with an alpha of .02 indicates that 56 participants are required to reach a power of .90.

#### 2.2.2 H1) Modifications in the global field power and/or topography of the event-related potentials to the “NoGo” trained cues

Given the nonlinearity of the relationship between the effect size of electrophysiological modulations and the associated behavioural modifications, a priori power calculations for our ERP hypotheses would not be relevant. Hence, the final sample size followed the power analyses from the behavioural HR, H2, and H3, especially given that these hypotheses require a sample size larger than those conventionally targeted in the ERP literature (Guttmann-Flury et al., 2019; Sands, 2009).

#### 2.2.3 H2) Training-induced modifications of implicit wanting of the “NoGo” trained cues

We determined the SESOI for the implicit wanting analyses to be a Cohen’s partial f of .25 (medium effect). A smaller effect would not be interpreted as positive even if they reach our significance threshold. A priori power analysis conducted with G-Power (Faul et al., 2007) for a 2 × 2 within-subject ANOVA with an alpha of .02 indicates that 56 participants are required to reach a power of .90.

#### 2.2.4 H3) Moderating effect of learning biases on the reduction in “NoGo” cues explicit liking

We determined the SESOI for the impact of the biases on the reduction in explicit liking of the “NoGo” items to be an R^2^ of 0.25 (medium effect size). A smaller effect would not be interpreted as positive even if they reach our significance threshold. A priori power analysis conducted with the pwrss R package (Bulus, 2023) indicated that an n of 51 was necessary to reach a power of .90 with an alpha of .02 for a linear model with a continuous outcome (explicit liking delta between pre- and post-training), a continuous moderator (learning regression coefficient of CS value on the eye-gaze index, weighting parameter *w*), and the baseline explicit liking as a covariate.

Overall, 56 participants were planned to be recruited to respect the most stringent hypotheses.

### 2.3 Recruitment and screening

The participants were recruited via public advertisement. The inclusion criteria included 18 to 45 years old healthy individuals with a BMI equal to or above 19; right-handed; the majority of the sugary drink types rated at least 3 out of 5 in our custom-made health questionnaire. The exclusion criteria were past or current diagnosis of eating disorders; current, planned pregnancy, or suspicion of pregnancy; previous, current, or planned diet; participation in a food executive control training study; any olfactory or gustative impairments; any visual or auditory impairment preventing the use of a smartphone video game.

### 2.4 Experimental design

Participants were emailed an information sheet to enable them to give informed consent at home, as well as a custom-made general health questionnaire for eligibility screening. After the eligibility screening, participants were asked to download our training software—The Diner—via the Android Appstore and fill out in-app analogue scales of items’ explicit liking. If the participants could not have access to the Android Appstore, they were lent a smartphone and went through the explicit liking questionnaire when coming to the laboratory.

Participants came to our laboratory at the University of Fribourg to complete either a sequential learning or a Pavlovian conditioning task (sequential learning/Pavlovian task chosen randomly). If the sequential learning task was done during the first visit, then the Pavlovian task would be done at the second visit and vice versa. They would then complete a Stimulus–Response Compatibility task (SRC), and an ERP GNG task. A 64-electrode EEG cap was installed to collect neural activity during the GNG task at both sessions. The SRC task was personalised to include the same “Go” and “NoGo” item categorisation as in the training software, as determined from in-app analogue scales of items’ explicit liking (see Section 2.5.4). In the ERP Go/NoGo task, each trained item was shown equally often in both “Go” and “NoGo” conditions, regardless of its original training category (e.g., a “Go” item during training appears as both “Go” and “NoGo” during the ERP GNG task).

Participants then completed 10 training sessions over a 2-week period (1 session per day; 12 min per session), with a gamified GNG task on the training software. The training only included the 60% most liked high-sugar drink items, as assessed by visual analogue scales. The terms “trained Go” and “trained NoGo” refer to the stimuli’s contingency during the training phase, which differed in the ERP Go/NoGo task. After training, the same in-app analogue scales of trained items’ explicit liking were filled by the participants.

Participants finally came back to the laboratory for a post-training session including either the sequential learning or Pavlovian conditioning task (whichever was not performed pre-training), and the same SRC and GNG tasks as during the pre-training session.

To reduce the influence of external variables on the outcomes of the task, participants were provided with a standardised offering of water and fruit before each visit. Additionally, the task sequence from the first visit was replicated during the second visit.

Participants were compensated with 135 CHF and received an additional small monetary reward corresponding to their gain in the sequential learning and Pavlovian task (ca. 15 CHF). Participants who did not complete the full training and/or the pre-/post-training measures were excluded from the study and compensated pro rata temporis.

### 2.5 Training task and pre-/post-training measures

#### 2.5.1 Stimuli generations

All pictures can be downloaded from our OSF study page (see Data and Code Availability section).

For the in-app analogue scales of explicit liking, SRC task (stimuli), ERP GNG task, and at-home GNG training, 57 pictures of sugary drinks were used. They represent the most popular sugary drinks (high sugar or sweetener rate) marketed in Switzerland, including 8 artificial energy drinks, 5 natural energy drinks, 4 iced coffee, 6 milky drinks, 16 citrus sodas, 3 colas, 2 other sodas, 2 kombucha, and 11 iced teas.

For the sequential learning and Pavlovian conditioning task, the character images and the fractals were generated using AI tools (see Supplementary Material).

#### 2.5.2 Analogue scales of explicit liking

The analogue scales of explicit liking were the same as in Najberg et al. (2021) and (2023) to capitalise on their robust findings. Within the training software, before and after the training, participants rated in a random sequence each sugary drink item between 0 (“not at all”) to 100 (“very much”) on analogue scales according to the question “Imagine drinking this, how much do you like it?”.

#### 2.5.3 Custom-made health questionnaire

To verify the eligibility criteria, a 14-item custom-made health questionnaire was used to assess the participants’ age, general health, recent weight loss or gain, dieting plans, participation in previous studies, and self-reported liking of the 9 identified sugary drinks categories (see Stimuli generation section) on a 5-item Likert scale. The questionnaire can be read on the study’s OSF page (see Data and Code Availability section).

#### 2.5.4 Categorisation of items as “Go” and “NoGo”

The “Go” items served as an active comparison condition to the “NoGo” items, matched on stimulus category and exposure. The trained items were the 60% most liked sugary drinks as measured during the pre-training in-app analogue scales of explicit liking. These selected items were then either categorised as “Go” (i.e., always prompting response execution) or “NoGo” (i.e., always prompting response withholding) items. This design ensured that the “Go” and “NoGo” items had an equivalent valuation and exposure during training.

To further prevent a generalisation of the effect of training between similar sugary drink pictures (e.g., Coca-Cola and Pepsi), sugary drinks of a similar type were always in the same GNG condition. To this end, we have identified nine types of sugary drinks within our picture dataset (see trained items section). During the calibration phase, a custom-made algorithm ensured that both the “Go” and “NoGo” conditions contained the same number of items and had an equivalent average liking within each participant. The detailed algorithm for categorising the sugary drinks as “Go” or “NoGo” is given in the Supplementary Material.

#### 2.5.5 Gamified Go/NoGo training

The GNG training task and its gamification followed the same procedure as Najberg et al. (2023), but without the neutral items, to capitalise on an already validated procedure.

To ensure response potency (i.e., a high pre-activation of motoric response) during the training, 70% of the trials consisted of “Go” items, and 30% of “NoGo”. This Go/NoGo proportion also strengthens the effect of the intervention, as the devaluation effect seems to appear when the “NoGo” rate is rare (Chen et al., 2016).

The reaction time threshold (RTT; i.e., the timing above which the participant is given a “Too late” feedback) of the “Go” items in this task was progressive. After six successful trials, the RTT increased by one level. The RTT is not challenging at first to result in plenty of successful trials, a parameter strengthening the devaluation effect, and then increases until the participant repeatedly fails the trials to both load the inhibitory control and maximize the engagement to the gamified training (see Supplementary Table S1 for difficulty level).

A demonstration of the video game is given on our OSF page (see Data and Code Availability section).

#### 2.5.6 ERP Go/NoGo task

A computerised, simplified version of the Go/NoGo training was executed in the laboratory for EEG recording. Participants were instructed to press as fast as possible on a response box to the items which were circled in green (“Go” trials, 60% of the trials) and withhold their response to the items circled in red (“NoGo” trials, 40% of the trials). The red and green cues were presented for 300 ms, and 100 ms post-stimuli’s onset. A total of 4 blocks of 200 trials each (120 “Go” and 80 “NoGo” per block; 800 trials total) were completed by the participants. Each trained item was shown equally often in both “Go” and “NoGo” conditions.

**Fig. 1.**
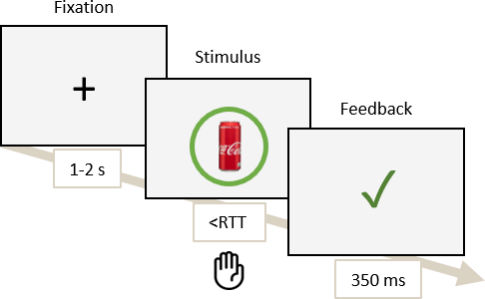
Schema of the Go/NoGo task describing the task timeline.

The RTT in this task is auto-adaptive, equaling 1.10 times the median of the task’s RTs and initialised after the first response, meaning that the first response is never given a “Too late” feedback. If a participant responded to a target item but was above the RTT, then the feedback “Too late” was displayed. If participants correctly withheld their response to a “NoGo” item (i.e., Correct Rejection) or responded to a “Go” item before the RTT (i.e., Hit), a green checkmark was displayed. If a participant erroneously responded to a “NoGo” item (i.e., False Alarm) or did not respond to a “Go” item (i.e., Miss), an “X” feedback was displayed. This procedure allowed for maintaining a stable level of difficulty across participants and blocks. The detailed experimental timeline is shown in Figure 1.

#### 2.5.7 Stimulus–Response Compatibility task

**Fig. 2.**
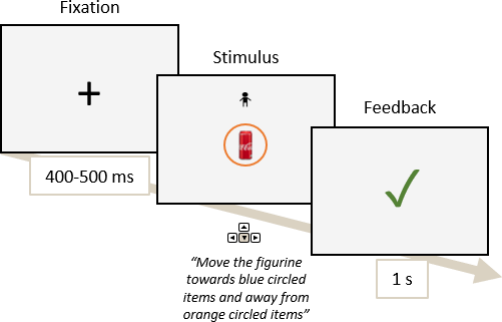
Schema of the Stimulus–Response Compatibility task describing the task timeline.

In the SRC task, participants were instructed to move a cartoon figurine (manikin) away from or towards sugary drink items based on a blue or orange cue (colour randomised across participants; Fig. 2) by repeated keyboard presses on “up” or “down” arrows. Participants were instructed to position their preferred finger between the arrows at the start of each trial. The character appeared pseudorandomly above or below the image. Both trained “Go” and “NoGo” items were evenly attributed to “away” and “toward” cues. The task consisted of 50 trials per cueing and item conditions, thus totalling 200 trials, and preceded by 20 practice trials.

The item and manikin disappeared as soon as the manikin reached the cued item or the edge of the screen and was followed by a feedback screen. The reaction time was computed at the first keyboard press.

#### 2.5.8 Pavlovian conditioning task

Participants completed a Pavlovian conditioning task (Schad et al., 2020) to assess their learning bias. The complete task consisted of the Pavlovian conditioning task and the forced choice task.

##### 2.5.8.1 Pavlovian conditioning

**Fig. 3.**
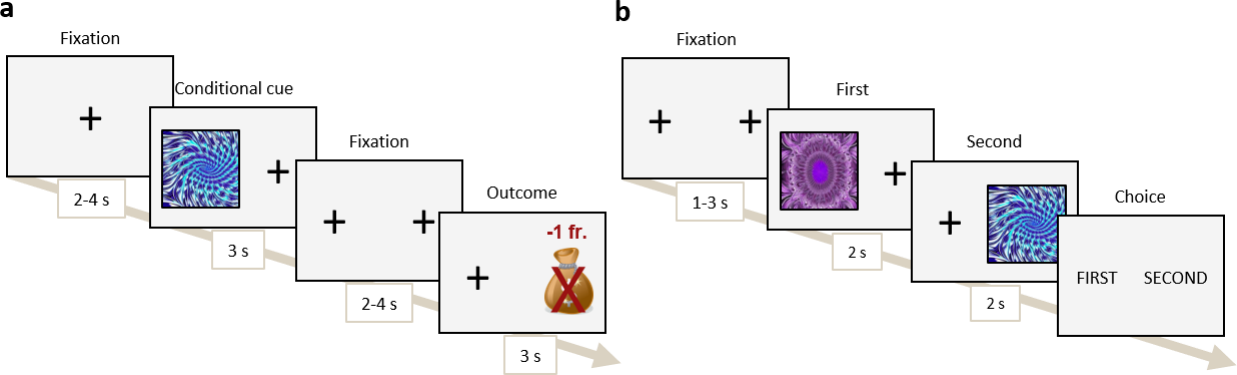
Schema of the Pavlovian conditioning task, describing the (a) Pavlovian conditioning and (b) Forced choice task timeline.

The Pavlovian conditioning task consisted of 80 trials, in which 5 fractal images (Conditional Stimulus; CS) were associated with 5 different reward values (Unconditional Stimulus; US): 2 negative values of -2 CHF and -1 CHF, 1 neutral value of 0 CHF, and 2 positive values of +1 CHF and +2 CHF. The identity of the fractal (CS) predicted the reward value (US). At each trial, a fractal stimulus was presented on the right or left side (randomised across trials) of the screen for 3 s, followed by two fixation crosses at the two potential stimulus locations during 3 s (Fig. 3). The reward value was then presented on the opposite side. Participants were instructed to observe the stimulus and memorise the fractal–value pairs.

We recorded the participant’s fixation time on the various locations (CS, US, and background) through the SMI RED 500 eye tracking system, recording binocularly at 500 Hz. The eye-gaze index was computed using the proportion of fixation time on the cue location minus the outcome location during the last second of the cue presentation. Details on the eye-tracking analysis pipeline and statistics are given in the Supplementary Material.

We measured the sign- or goal-tracking bias by extracting the regression coefficient of a linear regression predicting the gaze index from the reward value of the CS based on the work of Schad et al. (2020). A higher learning regression coefficient signifies a larger effect of the CS value and thus a higher sign-tracking bias.

##### 2.5.8.2 Forced choice task

To assess whether the Pavlovian learning took place during the previous phase, participants then performed a forced choice task of 30 trials in which they had to choose the CS with the highest associated value between 2 sequentially presented CSs. They were instructed to choose the fractal with the highest value and that they would receive 10% of the reward value associated with the chosen CS. Stimuli were presented one at a time for 2 s, after which the participants selected the first or second stimulus. Each possible CSs pairing was presented three times in a randomised order. Participants were asked to respond faster if their response was above 2 s.

#### 2.5.9 Sequential learning task

**Fig. 4.**
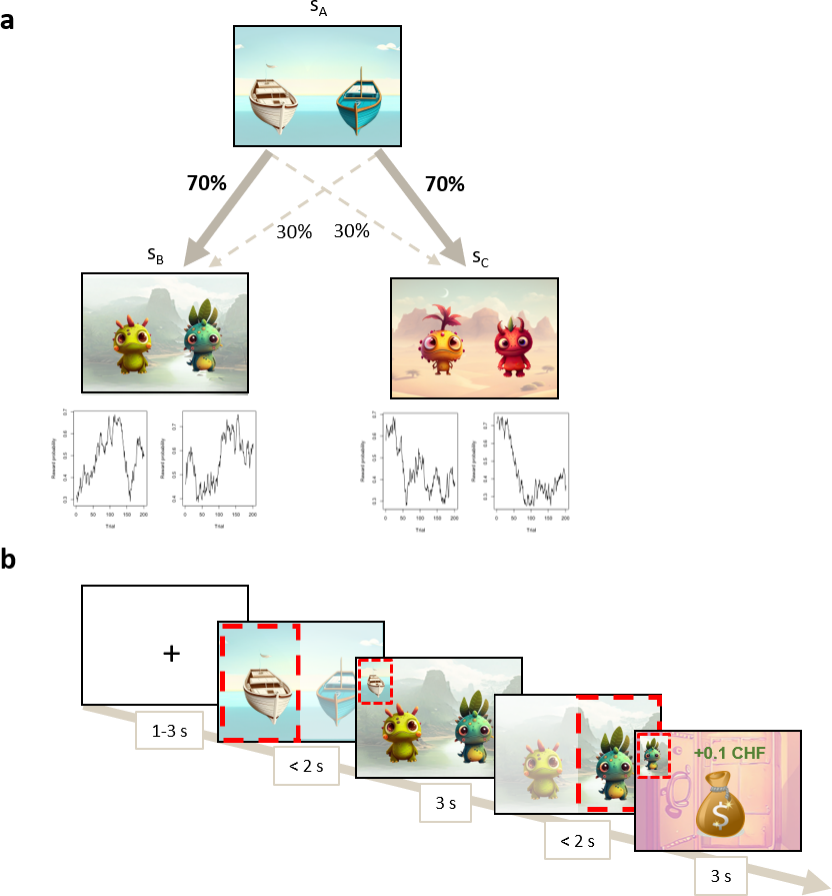
Schema of the sequential learning task, describing (a) the first choice between two boats at s_A_, each boat led preferentially to one of the two islands s_B_ or s_C_. Each monster on the islands led to a reward with a varying probability. (b) The sequential learning task timeline.

The sequential learning task consisted of 201 trials, subdivided into blocks of 67 trials with breaks. Participants underwent extensive instructions about the task before starting the experiment. Each trial consisted of two subsequent stages with choices. At stage 1, participants chose between a white and a blue boat, with each boat leading to one island of the two islands with a fixed probability (p = .7 or p = .3). At stage 2, participants chose between two monsters, with the choice of each monster being associated with a reward probability varying across trials. To encourage ongoing update of choice value, the probability of reward associated with each monster varied across the experiment following a random Gaussian walk (boundaries at 0.25 and 0.75, mean 0, SD 0.025) generated for each monster (cf. Fig 4a; Daw et al., 2011). The trial started with a 1–3 s fixation cross, followed by a 2 s window for the first choice, island presentation for 3 s and 2 s window for the stage 2 choice.

The position of the boats and monsters was pseudorandomly (p = .5) permuted between the left and right of the screen across trials. The Gaussian random walk curve was constant across participants, and the boat–island association probability varied pseudorandomly (p = .5) between participants.

Reinforcement learning computational modelling was used to calculate the tendency to rely on model-free over model-based learning strategies. The value of the participant’s first choice’s action was a weighted combination of the model-based and model-free computational models. The weighting parameter *w* of this equation was used as the index of the tendency to rely on model-free learning: a low *w* indicated a stronger reliance on the model-free strategy (cf. Daw et al., 2011; Voon et al., 2015; Supplementary Material for detailed computations, Supplementary Table S3 for parameter estimation, and Supplementary Fig. S2 for the parameter recovery analysis).

#### 2.5.10 Electrophysiological analyses

##### 2.5.10.1 Data collection

The 64-channel electroencephalogram was recorded at a sampling rate of 1,024 Hz with a Biosemi ActiveTwo system referenced to the common mode sense-driven right leg (CMS-DRL) ground placed on each side of the POz electrode. This circuitry consisted of a feedback loop driving the average potential across the montage as close as possible to the amplifier zero (cf. the Biosemi website for a diagram). The Cartool software (Brunet et al., 2011) was used for visualisation and importation/exportation of the raw and processed EEG data.

##### 2.5.10.2 Event-related potentials (ERP) pre-processing

The pre-processing followed the EEGlab-based (Delorme & Makeig, 2004) autoERP toolbox (Najberg et al., 2020). This pipeline would (1) reference the raw data to Cz, apply band-pass filtering between 0.5 and 40 Hz, (2) remove sinusoidal noise at 50 and 100 Hz (Cleanline, https://www.nitrc.org/projects/cleanline), (3) filter non-sinusoidal noise using Artifact Subspace Reconstruction (Chang et al., 2018; Mullen et al., 2015), (4) remove blinks detected during the stimulus onset (BLINKER plugin; Kleifges et al., 2017), (5) epoch the data to 100 ms before onset to 700 ms after onset, (6) apply baseline correction to correct for any remaining signal drifts, (7) interpolate identified bad channels using multiquadric interpolation (EEGInterp; Jäger & Buhmann, 2018), (8) exclude epochs with jumps of more than 30 µV from one-time frame (TF) to the next in at least one electrode, (9) exclude epochs with at least one TF with a voltage larger than 80 µV in an electrode, (10) average the epochs for each participant to create the ERPs, (11) re-reference the ERPs to the common average reference.

The targeted epochs were successful “NoGo” trials (i.e., correct rejection trials) at the GNG task because they were not contaminated by a motor response. All trials excluded during behavioural data pre-processing were also excluded from the EEG analyses (see Analysis plan section).

##### 2.5.10.3 Cluster-based component segmentation

A topographic temporal segmentation approach using the Cartool toolbox (Brunet et al., 2011) was used to detect the period of interest related to the occurrence of the ERP components used to confront our hypotheses. Group-averaged ERPs were fed to a hierarchical clustering based on an atomise and agglomerate approach (Brunet et al., 2011; Murray et al., 2008). The optimal number of clusters that explained best the grand-average datasets across conditions was identified using a modified version of the cross-validation criterion and the Krzanovski-Lai criterion (Tibshirani & Walther, 2005; see also Murray et al., 2008). This approach was based on evidence that the ERP map topographies did not vary randomly in time, but remained quasi-stable over 20–100 ms functional microstates—that is, the ERP components, before rapidly switching to other stable periods. This results in a data-driven protocol detecting the ERP components’ onset and offsets for all conditions (see Fargier & Laganaro, 2016; Laganaro et al., 2012; Najberg, Rigamonti, et al., 2021 for similar approaches).

Once segmented, the components were time locked to their GFP’s peak for the subsequent inferential analyses.

##### 2.5.10.4 ERP strength and topographic dissimilarity measures

The global ERP analyses have already been extensively described in our previous publications (Najberg, Wachtl, et al., 2021; Wicht et al., 2022). We provide only the essentials here. The global field power (GFP; Koenig & Melie-García, 2010; Koenig et al., 2011) and global map dissimilarity indices (GMD; Lehmann & Skrandies, 1980) were used as the primary outcome of the ERP analyses (H1).

The GFP indexes the strength of the electric field at the scalp and was defined as the spatial standard deviation of the field potentials (i.e., the root mean square of the difference between two normalised vectors computed across the entire electrode set). A large GFP means a stronger electric field arising from an increase in either the synchronisation or the extent of the neural sources underlying the scalp-recorded activity (Michel & Murray, 2012).

The GMD indexes differences in the topography of the electric field at the scalp, and was calculated as the root mean square of the difference between the potentials measured at each electrode for the different experimental conditions normalised by instantaneous GFP. Because changes in topography forcibly follow from changes in the configuration of the underlying active sources (Lehmann & Skrandies, 1980), topographic modulations reveal when distinct brain networks are activated across experimental conditions.

Since the GFP is insensitive to spatial (i.e., topographic) change in the potential distribution, and GMD is calculated on GFP-normalised data, the GFP and GMD are orthogonal measures that may vary independently. Either GFP or GMD modulations would index the neuroplastic changes induced by the intervention.

### 2.6 Analysis plan

All tests were computed using R base functions if not specified otherwise.

Regarding data exclusion, trials with RTs below 200 ms were excluded for all tasks. Dropouts and participants with missing data were not considered in their respective analyses and, thus, were replaced if this meant dropping below the planned sample size. Participants tagged as distribution outliers (see sub-sections below) were not replaced, as their exclusions aimed to improve the quality of the distribution.

Regarding the null hypothesis, Bayes Factors (BF_01_) between the model with and without the term of interest were computed using the BayesFactor R package (Morey & Rouder, 2018) following the method of Rouder et al. (2012) for all behavioural analyses. Only in case of non-significant results (p > .02) were they interpreted as support to the null hypothesis.

#### 2.6.1 HR) Training-induced modifications of explicit liking of the “NoGo” trained cues

First, to ensure a thorough filling of the analogue scales, we excluded items with less than 300 ms of response time.

Then, participants who were outside a 2.5*Median Average Deviation (MAD) range around the median of the explicit liking at either pre- or post-training were tagged as distribution outliers and removed from this analysis.

Finally, we assessed the modification in the participants’ average explicit liking of the “NoGo” trained items with the double interaction term of a Session (pre-, post-training) x Item Category (“Go”, “NoGo”) within-subject ANOVA. We expected a larger reduction of explicit liking for “NoGo” than “Go” items at the post- compared with the pre-training session.

The ANOVA was performed with the rstatix R package (Kassambara, 2023) and reported alongside its partial Cohen’s f. The homoscedasticity assumption was verified using the Levene test of the R car package (Fox & Weisberg, 2018). If violated, Greenhouse–Geisser correction was planned.

#### 2.6.2 H1) Modifications in the global field power and/or topography of the event-related potentials to the “NoGo” trained cues

First, we rejected participants not participating in the task or not understanding its rules, as indexed by a rate of False Alarm above 70% or a rate of “Miss” above 20% at either the pre- or post-training GNG task (threshold based on our previous publications; Hartmann et al., 2016; Najberg, Rigamonti, et al., 2021). Furthermore, we excluded participants without ERP in their post-processed signal, as determined by visual inspection.

Then, for each segmented ERP component, modulations of the global field power (GFP) and global map dissimilarity indices (GMD) of the electric field at the scalp level during Correct Rejection responses of the in-lab GNG tasks were assessed with the double interaction term of a within-subjects Session (pre-, post-training) x within-subjects Item Category (Go, NoGo) at each TF using ANOVA-like non-parametric randomisation statistics (i.e., Monte Carlo bootstrapping). These analyses were performed using the free toolbox RAGU (Koenig et al., 2011).

This hypothesis focused primarily on the effect of the GNG training on the “NoGo” items. Adding the “Go” items as a level in the Item Category factor allowed us to rule out an effect of mere exposure to the stimuli in the interaction term. Yet, to prevent a change in the ERP to the “Go” items from driving the interaction term, if a pre- vs. post-training effect on the GFP and GMD analyses on the “Go” item category was observed, then the Session x Item Category double interaction would be reduced to a pre- vs. post-training contrast on the “NoGo” items only.

An ERP modulation was considered for interpretation only if at least 12 consecutive TFs showed a significant interaction in a segmented component (see component segmentation section). Because any changes in either GFP or GMD were considered as evidence for training-induced plastic changes, the alpha threshold was set to .01 to correct for multiple comparisons.

#### 2.6.3 H2) Training-induced modifications of implicit wanting of the “NoGo” trained cues

The implicit wanting for an item is calculated as the difference in reaction times between the participants moving away and towards the trained item (i.e., IW = RT.Away – RT.Toward). The more positive this value, the more the item is implicitly liked.

First, participants who were outside a 2.5*MAD range around the median of the implicit wanting at either pre- or post-training were tagged as distribution outliers and removed from this analysis.

Then, we assessed the modification in the participants’ average implicit wanting of the “NoGo” trained items with the double interaction term of Session (pre-, post-training) x Item Category (“Go”, “NoGo”) within-subject ANOVA. We expected a larger reduction of implicit wanting for “NoGo” than “Go” items at the post- compared with the pre-training session.

The ANOVA was performed with the rstatix R package (Kassambara, 2023) and reported alongside its partial Cohen’s f. The homoscedasticity assumption was verified using the Levene test of the R car package (Fox & Weisberg, 2018). If violated, the Greenhouse–Geisser correction was planned.

#### 2.6.4 H3) Moderating effect of learning biases on the reduction in “NoGo” cues explicit liking

First, to ensure a thorough filling of the analogue scales, we excluded items with less than 300 ms of response time. We set a minimally relevant pre–post reduction (i.e., Cohen’s d ≥ 0.4) in the explicit liking of the “NoGo” items to be observed to continue testing this hypothesis. No results would have been interpreted for this hypothesis otherwise.

Second, the participants’ average explicit likings at pre- and post-training were subtracted from each other to create an explicit liking delta.

Third, participants with an error rate above 10% in the Forced Choice task were excluded from the analysis. Participants who could have been excluded in this way would have been replaced if this meant dropping below the planned sample size. Additionally, participants who were outside a 2.5*MAD range around the median of the explicit liking delta and learning biases (H3a: regression coefficient of CS value on the eye-gaze index for Pavlovian Learning; H3b: *w* index for the sequential learning) were considered distribution outliers and removed from this analysis.

Finally, we computed a linear mixed model with the explicit liking delta as the outcome, the learning bias (H3a: regression coefficient of CS value on the eye-gaze index for Pavlovian Learning; H3b: *w* index for the sequential learning) as the variable of interest, and with the baseline explicit liking as a random effect (i.e., covariate). We expected that a larger sign-tracking/model-free bias would predict a larger reduction in explicit liking. The baseline explicit liking was used as a covariate to control for the bias that a larger baseline explicit liking would allow more room for a pre–post delta.

This model was computed using the lm function in R.

Both learning biases were conceptualised as stable individual traits and were, therefore, assessed once per participant, either before or after training. This assumption was verified by testing the effect of visit order on learning bias scores, which revealed no significant association (p-values ≥ .263; see Supplementary Material for detailed analyses and results).

## 3 Results

### 3.1 Participants

A total of 286 participants completed the consent form online, and 136 respected the inclusion criteria. Sixty-one participants were recruited for the study, and 1 participant dropped out before the second visit, resulting in a total of 60 participants (see Supplementary Fig. S1 for sample size progression, and Table 2 for participants’ characteristics).

**Table 2.** Participants’ characteristics.

Mean ± SD	Participants (n = 60)
Age	25.2 ± 4.3
Gender Ratio (F:M)	0.67 (40F:20M)
Body Mass Index	23.35 ± 4.6
Baseline explicit liking	69.1 ± 14.0

### 3.2 HR) Training-induced modifications of explicit liking of the “NoGo” trained cues

**Fig. 5.**
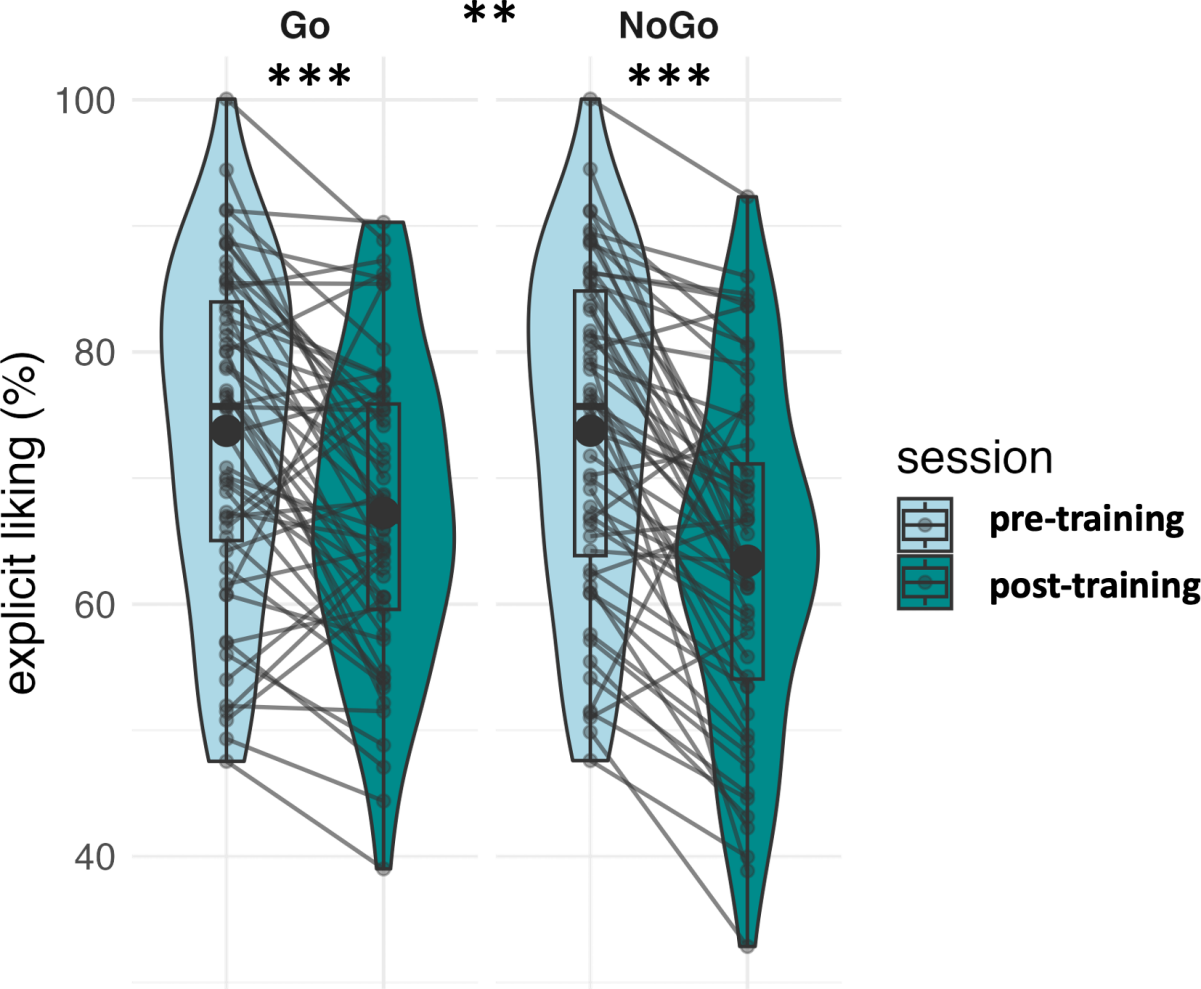
Representation of explicit liking ratings (%) at pre- and post-training for items trained as “Go” and “NoGo”. Individual data points (semi-transparent dots), means (bold dot), distributions’ density (violin), medians, first and third quartiles (horizontal bars), and the 1.5 inter-quartiles range (whiskers) are represented. **: p < .01, ***: p < .001.

Results of the sugary drink explicit liking are reported in Supplementary Table S7 and Figure 5.

Three participants were lost due to technical issues, one distribution outlier was excluded. In total, 56 participants were included in the analysis.

For the Condition x Session interaction on sugary drinks’ explicit liking, the homoscedasticity assumption was respected (Levene’s Test: F[3, 220] = 0.28, p = .838), thus no correction was applied. The double interaction term was significant (F[1,55] = 8.41, p = .005, BF_01_ = 0.81), and above our minimal effect size of interest (partial Cohen’s f = 0.39).

### 3.3 H1) Modifications in the global field power and/or topography of the event-related potentials to the “NoGo” trained cues

One participant was excluded due to a high rate of “Miss” in the GNG task, and one did not visually present an ERP. In total, 58 participants were included in the analysis.

The processed ERPs were satisfactory, with around 146 epochs included in the ERP on average (see Supplementary Table S4 for EEG pre-processing results). The P1 and N2 were segmented for each condition and then cut to have one interval to analyse per ERP component locked across all conditions at their respective GFP’s peak. This resulted for the P1 in a 38 ms component with the GFP’s peak at around 85 ms, and for N2 in an 82 ms component, with the GFP’s peak at around 222 ms. The P1 and N2 segmentation intervals’ details are given in the Supplementary Material.

**Fig. 6.**
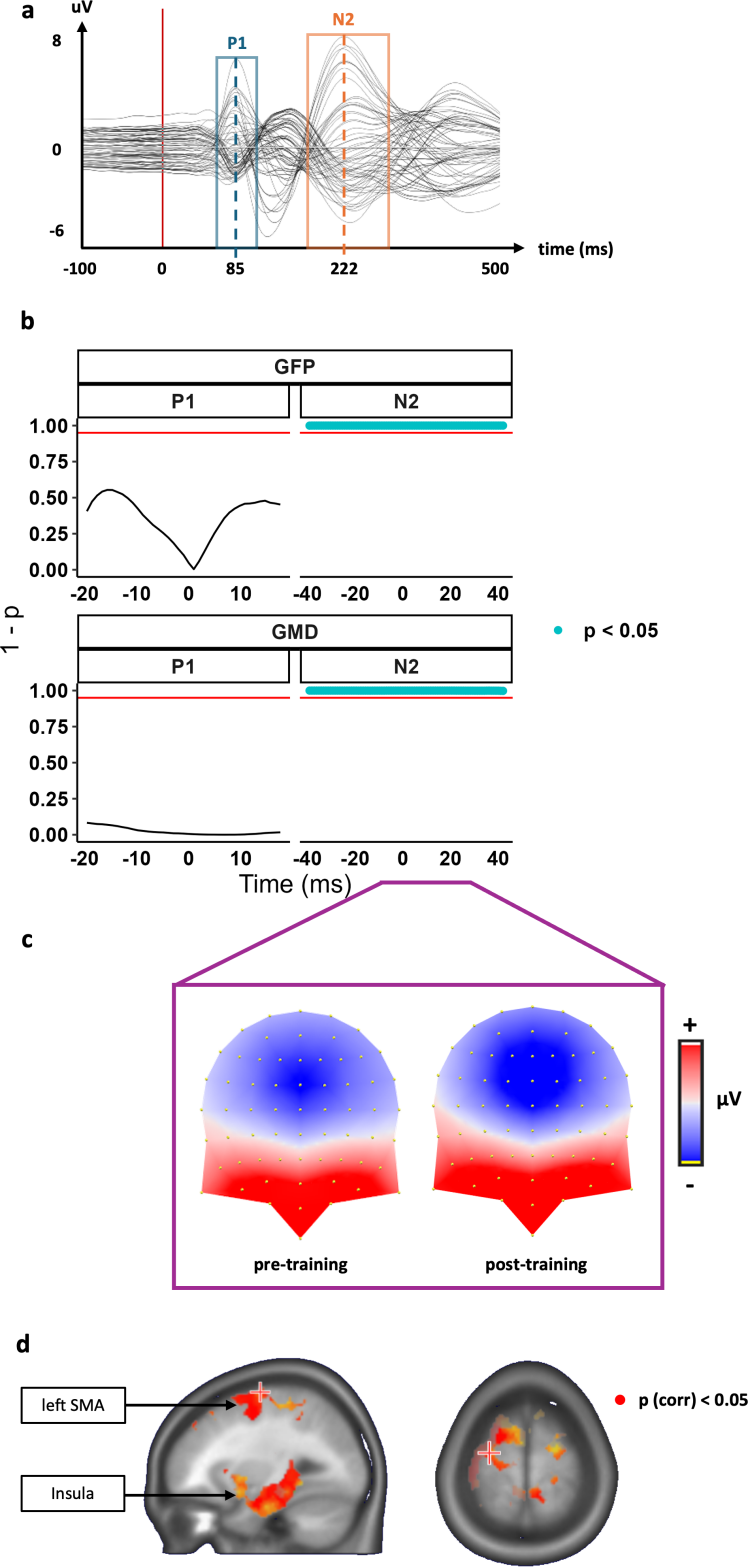
Representation of ERP responses and analyses for the trained “NoGo” condition. (a) Example of a grand-average ERP of one condition: pre-training “NoGo”. The amplitude (y-axis), the time (x-axis), the event onset (red vertical bar), and both components’ interval (P1: blue square, N2: orange square) are represented. (b) Inverted p-values (y-axes; blue dot when significant) are represented at each ms (x-axes) for both P1 and N2 ERP components (left and right columns) and for both the global field power (GFP) and global map dissimilarity indices (GMD) analyses (first and second rows). The alpha threshold (.05) is represented with a red horizontal line. (c) ERP topography over the period of the GFP-locked N2 component (ca. 177–269 ms). Positive (red) and negative (blue) amplitudes are represented at the scalp level. (d) Distribution of p-values (red areas) resulting from source-estimation analyses (pre- vs. post-training) represented on an average MRI brain scan during the GFP-locked N2 component (ca. 177–269 ms).

Results of the ERP analyses are reported in Figure 6.

As registered, for P1, because the effect of training had no significant effect on the Trained “Go” items, analyses on this component were assessed using the interaction term of a 2 (pre- vs. post-training) x 2 (Trained Go vs. Trained NoGo). For N2, because the effect of training presented a significant effect on the Trained “Go” items, analyses on this component were assessed with the pre- vs. post-training on the Trained “NoGo” items only. See Supplementary Tables S5, S6, and Supplementary Fig. S3 for detailed results on these analyses.

For P1, there were no significant TF at any point for both the GFP and GMD, resulting in P1 presenting no change towards the trained “NoGo” items after training (Fig. 6b).

For N2, there were 84 consecutive significant TFs spanning the whole component for both the GFP and GMD (Fig. 6b). In terms of strength, this is driven by greater negative activity in the centro-frontal electrodes post-training, and in terms of topography, this is driven by a difference in the left temporal electrodes presenting positive activity post-training.

Source estimation during the period of interest (see Supplementary Material for detailed method) indicates changes in both the supplementary motor area (SMA; Fig. 6d) and the insula (Fig. 6d) during N2.

### 3.4 H2) Training-induced modifications of implicit wanting of the “NoGo” trained cues

Eight distribution outliers were excluded. In total, 52 participants were included in the analysis.

**Fig. 7.**
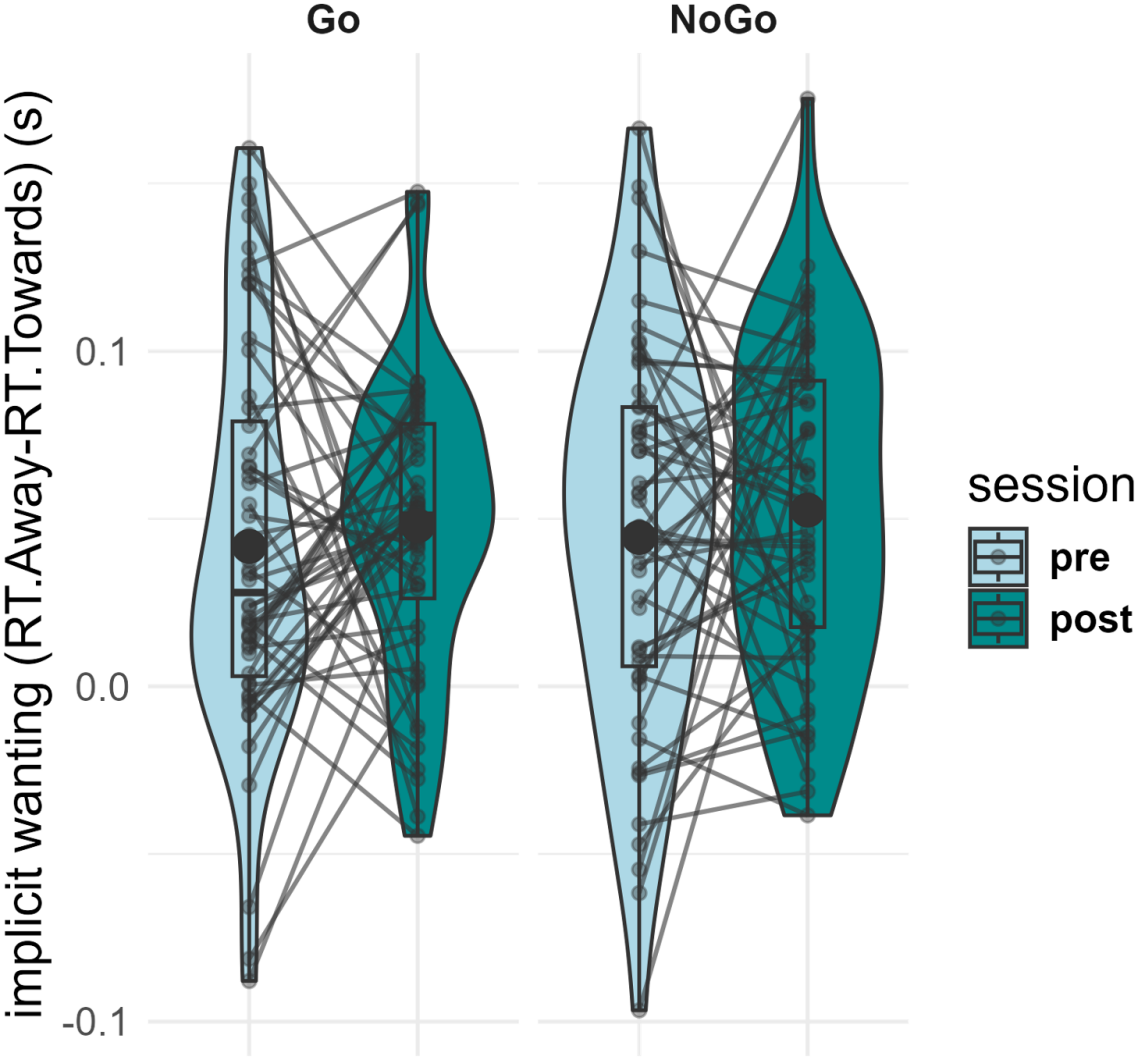
Violin plot representing implicit wanting at pre- and post-training for items trained as “Go” and “NoGo”. Individual data points, means (bold circle), distributions’ density (violin), medians, first and third quartiles (horizontal bars), and the 1.5 inter-quartiles range (whiskers) are represented.

Results of the implicit wanting analysis are reported in Supplementary Table S8 and Figure 7.

For the Condition x Session interaction on sugary drinks implicit wanting, the homoscedasticity assumption was respected (Levene’s Test: F[3, 204] = 1.82, p = .144), thus no correction was applied. The double interaction term was not significant (ANOVA: F[1,51] = 0.013, p = .910) and below our minimal effect size of interest (partial Cohen’s f = 0.02), with its Bayes Factor providing moderate evidence supporting the absence of an effect (BF_01_ = 5.44).

### 3.5 H3) Moderating effect of learning biases on the reduction in “NoGo” cues explicit liking

Three participants were lost due to technical issues resulting in no explicit liking VAS. In total, 58 participants were included in the analysis. We observed a pre–post reduction in explicit liking above our minimally relevant threshold (Cohen’s d = 0.78), and could thus continue with the analysis.

**Fig. 8.**
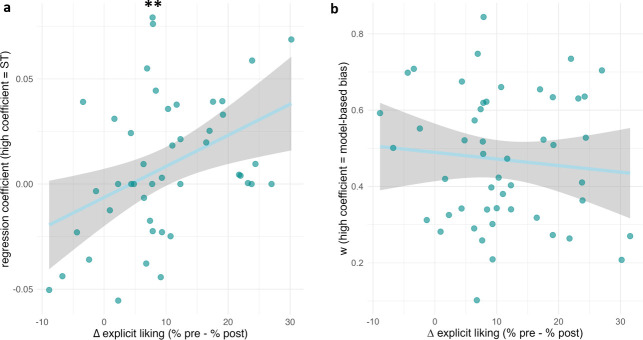
Learning bias and delta explicit liking (% pre- % post-training) affective learning bias as (a) Pavlovian bias, (b) *w* (high = model-based bias). Individual data points (coloured dots), regression line (coloured line) and 95% confidence interval (grey area) are represented. **: p < .01.

Results of the effect of learning bias on the reduction of explicit liking are reported in Supplementary Table S9 and Figure 8.

#### 3.5.1 H3a) Learning bias as sign-tracking bias

Two participants were lost due to technical issues in the Pavlovian conditioning task. Two participants were excluded due to a high error rate in the Forced Choice task. Eight distribution outliers were excluded, the analysis thus included 45 participants.

We observed a significant association of the Pavlovian bias with the devaluation (β = 107.94, SE = 38.70, t[42] = 2.79, p =. 008, BF_01_ = 0.141), with an R^2^ of 0.21, suggesting that a stronger sign-tracking bias is associated with a greater devaluation effect.

#### 3.5.2 H3b) Learning bias as model-free bias

One participant was lost due to technical issues in the sequential learning task. Two distribution outliers were excluded, the final analysis thus included 54 participants. The distribution of inferred free parameters is given in Supplementary Table S3.

We did not observe a significant effect of the bias *w* (i.e., low *w* indexing reliance on model-free strategy) on the devaluation (β = -7.20, SE = 6.84, t[51] = -1.05, p = .297), with an R^2^ of 0.01 and its Bayes Factor (BF_01_ = 1.83) providing weak evidence supporting the absence of an effect, and suggesting there is no meaningful association between the model-free bias and the devaluation.

## 4 Discussion

We conducted a within-participant pre–post intervention study to assess the effects of a 10-session gamified food Go/NoGo (GNG) training. We hypothesised that the training would reduce the explicit liking of the “NoGo” trained cues (HR), that it would modulate the P1 and/or N2 event-related potentials to “NoGo” cues (H1), that it would reduce implicit wanting for “NoGo” cues (H2), and that individual learning biases (indexed as Pavlovian and model-free over model-based biases measured in a Pavlovian conditioning and sequential learning task, respectively) would moderate the training’s efficacy. Importantly, without untrained stimuli or a no-training control group, any observed pre–post effects cannot be uniquely attributed to the intervention. Accordingly, our inference primarily concerns differential changes between “Go” and “NoGo” trained items.

### 4.1 Training-induced change of explicit liking of the “NoGo” trained items

We confirmed our hypothesis for a larger decrease in the explicit liking of trained “NoGo” than “Go” items, consistent with previous findings on GNG training effects on food devaluation (Najberg, Rigamonti, et al., 2021; Najberg et al., 2023). Since we used the same item category (i.e., sugary drinks) in both “Go” and “NoGo” conditions, the stronger devaluation observed for “NoGo” items suggests that response training effects were item specific and did not generalise to the broader sugary drinks category. This suggests mechanisms tied to the specific stimulus–response associations rather than conscious identification of contingencies. Interestingly, while larger for “NoGo” items, both “Go” and “NoGo” trained items showed a reduced valuation, which contrasts with previous findings where “Go” items often show stable or increased valuation (Najberg, Rigamonti, et al., 2021; Najberg et al., 2023). This may be due to an expectancy effect: unlike previous studies where “Go” items were healthy and “NoGo” items were unhealthy, in our design, both categories consisted of unhealthy items; as a result, participants may have expected the intervention to reduce the value of all unhealthy stimuli, extending the effect to “Go” items as well.

### 4.2 Training-induced change in the global field power and topography of the event-related potentials to the “NoGo” trained cues

At the neurophysiological level, we first examined training-induced changes in the P1 ERP component to assess whether food response training reduces cue valuation via a reduction of early stage attentional responses to the trained cues (H1a). For this component, we did not find an influence of the training on either the global field power (GFP) or global map dissimilarity (GMD) of the correctly rejected “NoGo” trained items’ ERPs during the GNG task. These findings suggest that training-induced devaluation did not follow from a change in early attentional saliency to the “NoGo” items, as indexed by the P1 component.

We then examined the frontocentral N2, an ERP component typically interpreted as reflecting conflict detection and resolution between automatic response tendencies and task demands (De Pretto et al., 2019; Folstein et al., 2008) (H1b). We observed training-induced modulations on trained “NoGo” items at the level of both the strength and topography of the scalp field potentials. The “NoGo” N2 GFP was stronger post- vs pre-training, indicating enhanced engagement of inhibitory processes in response to the trained rewarding stimuli. The topographic modulation indicates that the training not only induced quantitative change in the strength of the response of the underlying neural generators, but also a change in their configuration: different brain networks were engaged to “NoGo” stimuli with training.

Our estimations of the brain sources of the modulations observed at the scalp during this period of interest (see Supplementary Material for detailed method) indicate that they follow from changes in both the supplementary motor area (SMA; Fig. 6) and the insula (Fig. 6) during N2. These findings are consistent with previous work indicating that GNG training affects cognitive control and sensorimotor activation (van de Vijver et al., 2018), and suggest that the devaluation depends on changes in motor execution and reward/motivational processing. The timing and nature of this ERP modulation support the hypothesis for an emergence of automatic inhibitory responses (Houben et al., 2012; Verbruggen & Logan, 2008) with Go/NoGo training, and the associative stop-learning account (Best et al., 2016; Veling et al., 2017; Verbruggen et al., 2014). These findings could also align with value-updating theories (Veling et al., 2008, 2022) as the N2 component was shown to be sensitive to food cue salience (Carbine et al., 2018), and insular activity reflects the multimodal integration and valuation of salient food cues (Frank et al., 2013). The co-occurrence of these neurophysiological changes with a reduction in explicit liking of “NoGo” items supports the idea that enhanced and reorganised inhibitory control contributes to cue devaluation.

### 4.3 Training-induced modifications of implicit wanting of the “NoGo” trained cues

We then investigated whether the training led to a larger decrease in the implicit motivational response of the target in the trained “NoGo” than trained “Go” items. The “implicit wanting” was measured with a Stimulus–Response Compatibility (SRC) task (Mogg et al., 2003; Tibboel et al., 2015), which indexes the incentive value of stimuli as a slowing down of (incongruent) avoidance motor responses to the wanted cues. Contrary to our hypothesis, we did not observe a larger decrease in the “NoGo” than in the “Go” items’ implicit wanting with training.

We observed a general pre–post decrease in SRC reaction times in both approach and avoidance responses (see Supplementary Material and Supplementary Fig. S4 for detailed analyses and results). This non-specific speed-up (e.g., practice or repeated testing) may have reduced sensitivity to detect condition-specific changes in implicit motivational responses. Additionally, our split-half reliability analysis showed relatively low reliability (rSB = 0.48; see Supplementary Material and Supplementary Fig. S5 for detailed analyses and results), indicating substantial noise in the measure. This limited reliability may have attenuated observable effect sizes and, therefore, provides a plausible methodological explanation for the observed null effect. Those results may also be due to the task design, as there are indeed concerns in the literature regarding the state dependency of approach-avoidance measures (Kahveci et al., 2020, 2021). Repeated measures of approach avoidance may thus be necessary to improve the reliability of such indexes (van Alebeek et al., 2024; Zech et al., 2023). Taken together, these considerations suggest that limitations in our design may explain the null result on implicit wanting.

### 4.4 Moderating effect of learning biases on the reduction in “NoGo” cues explicit liking

To test the hypothesis that shared cognitive mechanisms underlie the effect of response training and learning, we examined whether changes in explicit liking were moderated by individual learning biases, as measured by a Pavlovian conditioning and a sequential learning task.

We found a moderating effect of sign-tracking bias on the devaluation of “NoGo” items, suggesting that individuals more prone to attributing motivational salience to cues showed stronger devaluation. Although the effect was statistically significant and of medium size (R^2^ = 0.21, p = .008), it fell below the threshold of our smallest effect size of interest (R^2^ = 0.25) and thus was not considered meaningful as per our preregistration criteria. Yet, the result hints at a role for Pavlovian learning biases in responsiveness to GNG training. We posited such overlap because Pavlovian learning plays a crucial role in eating behaviour, as do item valuation: since food-related cues indeed acquire motivational salience through associative learning processes (Boswell & Kober, 2016; Hill, 2007; Jansen, 1998; Martin-Soelch et al., 2007; Sun & Kober, 2020), appetitive food cues are associated with a tendency to respond, whereas non-appetitive cues are associated with a tendency not to respond (Ereira et al., 2021; Guitart-Masip et al., 2014). According to Veling et al. (2022), during GNG training, prediction errors might arise when task demands (e.g., inhibiting response to an appetitive stimulus labelled as “NoGo”) conflict with those hardwired Pavlovian tendencies to approach, leading to a reassignment of value manifesting as the item devaluation (Veling et al., 2022). In this context, the “Go” and “NoGo” signal (i.e., coloured frame) constitutes the immediate action–demand cue, and the initially appetitive food cues’ value will gradually decrease as it becomes associated with the requirement to withhold action. As a result, individuals exhibiting a sign-tracking bias (i.e., attributing motivational salience to the cue itself) are more sensitive to a decrease in that cue’s incentive value, ultimately leading to larger devaluation effect. The medium size of the effect suggests that this may be only one of several mechanisms influencing responsiveness to GNG training. Future studies may integrate additional sensitive measures of cue valuation (e.g., approach bias; Zech et al., 2023) or implicitly measured liking (Tzavella & Chambers, 2023) to disentangle the involved components.

We found no moderating effect of the model-free bias on the devaluation of “NoGo” items. This suggests that individuals relying more on model-free over model-based learning were not more sensitive to the devaluation effects of GNG training in our experimental setting. Reinforcement learning is defined as the process through which environmental cues acquire value and influence behaviour (Pavlov (1927), 2010). Within this framework, sequential learning is more closely tied to action selection mechanisms and may not be directly reflected in stimulus valuation processes. Accordingly, this effect may be observable only in action-related outcomes. To clarify this question, future research should investigate the impact of GNG training on outcomes involving action selection (e.g., food choices and their consequences, such as weight), rather than on subjective evaluations of stimulus value.

These results suggest that GNG training likely shares mechanisms with Pavlovian conditioning through cue valuation processes, whereas reinforcement learning, being more closely tied to action selection, does not appear to underlie “NoGo” items devaluation.

## 5 Conclusion

This study aimed to understand mechanisms underlying a response training intervention. We replicated previous findings showing that a gamified Go/NoGo training decreases the explicit liking of targeted stimuli. At the neurophysiological level, early stage attentional responses to the trained cues, as measured with the P1 ERP component, remained unchanged after the training, suggesting that if a reduction in valuation occurs, it may not rely on early attentional processing. We observed modulations in the strength and topography of the N2 ERP component indexing conflict detection and resolution, consistent with the emergence of “inhibition reflexes” and devaluation through the active suppression of approach tendencies. We found no evidence for a decrease in implicit motivational responses, which might reflect the absence of training effects on implicit wanting or limitations in task design. We found that sign-tracking bias, but not model-free bias, moderated explicit devaluation, pointing to Pavlovian cue-valuation processes as a driver of sensitivity to GNG training. Altogether, our findings identify neural and cognitive components of response training, supporting its potential for targeted, mechanism-based interventions against maladaptive behaviours triggered by reward cues.

## Supplementary Material

Supplementary Material

## Data Availability

The data and analyses scripts are available on our OSF page: https://osf.io/y49gv.

## Stage 1 and Stage 2 Manuscripts

The Stage 1 manuscript as recommended by PCI-RR is given here: https://osf.io/jgr9z/files/osfstorage, and the Stage 2 recommended manuscript is given here: https://osf.io/wdy4u.

## Supplementary Materials

The Supplementary material is given on our OSF page: https://osf.io/zbu52. Supplementary material for this article is given here: https://doi.org/10.1162/IMAG.a.1180#supplementary-data.
