# Supplementary material for: Plastic brain mechanisms supporting reduction in cravings induced by response training
Source: Imaging Neurosci (Camb). 2026 Mar 30;4:IMAG.a.1180. doi: 10.1162/IMAG.a.1180 (PMC13037660; doi:10.1162/IMAG.a.1180)
Supplement: Supplementary Material [file IMAG.a.1180_supp.pdf]

Supplementary material for Stage 2 RR: "Plastic brain mechanisms supporting reduction in cravings induced by response training"

1. Stimuli generation

1.1 Sequential learning

The stimuli of the task consist in boats and monsters on sea and islands backgrounds. All images have been generated using the AI image generator tool Midjourney. All images have been generated using the AI image generator tool Midjourney.

Boat prompt: "Two similar wooden boats standing straight next to each other in the same ocean, exact same size and orientation, one is blue and the other one is white, cartoon style."

Monsters prompt: "Two cute cartoon rainforest monsters, smiling, in different colours, standing completely straight, distant from each other, same size, looking at us, lilo et stitch style."

Desert monsters: "In the same cartoon style as (Rainforest monsters image) two cute cartoon rainforest monsters, smiling, in different colours, standing completely straight, distant from each other, same size, looking at us"

After generating different sets of images using variations of these prompts, two boats and four monsters with similar styles and no obvious positive or negative connotation were selected.

Images were then edited using Photoshop and AI clean up tools to simplify the monsters' and the boats appearance.

Backgrounds were extracted from generated images of rainforest monsters, desert monsters and boats and edited to remove the monsters and boats, as well as most of the original features.

Backgrounds for reward and no reward were generated using DALL·E 2, with the prompt "collection of interiors of a bank safe, one in warm colours, the other one is in cold grey colours in cartoon style". Images were then simplified using AI clean up tools and colours were adjusted using Photoshop.

1.2 Pavlovian conditioning and Pavlovian-to-Instrumental transfer

Fractals images have been generated using the AI tools DALL·E 2 and Stable Diffusion.

Prompts for fractals: "Collection of beautiful fractals in different colours."

After generating different sets of images using variations of these prompts, five fractals with no obvious positive or negative connotation were selected.

The reward image was chosen from Pixabay with the keyword "money bag" (Pixabay, n.d., https://www.pixabay.com)

2. Gamified Go/NoGo training

2.1 Algorithm categorising the sugary drinks as "Go" or "NoGo"

During the calibration phase, we implemented the following custom-made algorithm to ensure that both the "Go" and "NoGo" conditions contained the same number of items and had an equivalent average liking within each participant:

1) While there are items still non-attributed to the “Go” or “NoGo” conditions, select the items of a random drink type category and attribute them to the condition with the least number of items.
2) Compute the average liking rate in both conditions. In the condition with the higher number of items, remove the items further away from the other condition’s average. For example, if there are three more items in the “Go” than in the “NoGo” condition, and the “Go” condition is less liked than the “NoGo”, then the three least liked “Go” items are removed.
3) Repeat the steps above 300 times and pick the result returning the smallest difference in liking between the “Go” and “NoGo” conditions, as assessed with a Cohen’s d.

2.2 Difficulty level

The reaction time threshold (RTT; i.e., the timing above which the participant is given a “Too late” feedback) of the Go items in this task is progressive. After 6 successful trials, the RTT increases of a level. The RTT is not challenging at first to result in plenty of successful trials, a parameter strengthening the devaluation effect, and then increases until the participant repeatedly fails the trials to both load the inhibitory control and maximize the engagement to the gamified training (Supplementary Table 1).

Difficulty level	1	2	3	4	5	6	7	8	9	10	11	12	13	14	15	16	17	18
RTT in seconds	1.1	1	.9	.8	.725	.675	.625	.575	.55	.525	.5	.475	.452	.43	.407	.387	.36	.33

Supplementary Table S1. Difficulty level of the gamified Go/NoGo training task.

3. Design table

Question	Hypothesis	Sampling plan	Analysis Plan	Rationale for deciding the sensitivity of the test for confirming or disconfirming the hypothesis	Interpretation given to different outcomes	Theory that could be shown wrong by the outcomes
Will the intervention decrease the explicit liking of “NoGo” trained items?	HR: A larger pre- post-training reduction of the explicit liking in the “NoGo” than “Go” items.	56 participants based on a priori power analysis.<br>The smallest effect size of interest is a Cohen’s partial f of .25.<br>Will be excluded during analyses the distribution outliers on the explicit liking at both sessions (above 2.5*MAD around the median).	If the homoscedasticity assumption is respected, then ANOVA with the within-subjects factors Session (pre-, post-training) and Item Category (“Go”, “NoGo”) on the explicit liking outcome. If the homoscedasticity assumption is violated, then the Greenhouse-Geisser correction will be used.	As the intervention showed a robust effect on explicit liking in both of our previous studies (Cohen’s d = 1.7 and 2.1 for Najberg et al., 2021 and 2023 respectively), we will not search for any effect smaller than a Cohen’s partial f of .25.	If $p<0.02$ on the double interaction term, then the intervention has a different effect on trained “NoGo” than trained “Go” items. In the case that $p > 0.02$, BF_{01} should be above 3 to support the absence of interaction.	If we are unable to replicate our previous findings, we conclude that differences in procedure between our last studies have disrupted the effect of the intervention (i.e., sugary drinks for both “Go” and “NoGo” items, duration of the training, explicit categorisation of stimuli[1,] etc).
Will we observe neural correlates supporting the hypothesised mechanisms of action of food response training?	H1a: Different pre- to post-training modulations of the GFP and GMD indexes during the correct withholding responses of items trained as “Go” and trained as “NoGo” during the intervention.<br><br>These differences should be expressed in the P1 ERP occipital component at 50-150ms post-stimuli onset, associated with attentional saliency. No direction in these modulations is expected.	56 participants based on behavioural rationales (see section 2.2).<br><br>Will be excluded during analyses:<br>- participants with an error rate above 70% on “NoGo” trials or above 20% on “Go” trials.<br>- No ERP on post-signal, as determined by visual inspection.	ANOVA-like non-parametric randomisation statistics with the within-subjects factors Session (pre-, post-training) and within-subjects Item Category (“Go”, “NoGo”) on the GFP and GMD indexes.	Non-applicable.	If $p<0.02$ on the double interaction term during at least twelve consecutive time frames (i.e., ca. 12ms for a sampling rate of 1024Hz) on either the GFP or GMD indexes, then the corresponding food response training’s mechanism of action is verified in a neuro-imaging setting.	If we fail to reject the null hypothesis, we could conclude that the response training intervention did not change the attentional saliency of items trained as “NoGo” compared to “Go”, which could indicate that the exposure effect is the main driver of the intervention’s effect.
	H1b: Same expectation in GFP and GMD					If we fail to reject the null hypothesis, we could conclude that

	modulations than H2a but expressed in the N2 ERP frontocentral component at 150-300ms post-stimuli onset, associated with motoric inhibition. No direction in these modulations is expected.					there are no changes in how trained “NoGo” items trigger inhibition with the response training intervention.
Will the intervention decrease the implicit wanting in “NoGo” trained items?	H2: A larger decrease in the “NoGo” than “Go” items’ implicit wanting between pre- and post-training.	56 participants based on a priori power analysis.<br>The smallest effect size of interest is a Cohen’s partial f of .25<br>Will be excluded during analyses the distribution outliers on the implicit wanting at both sessions (above 2.5*MAD around the median).	If the homoscedasticity assumption is respected, then ANOVA with the within-subjects factors Session (pre-, post-training) and Item Category (“Go”, “NoGo”) on the implicit wanting outcome. If the homoscedasticity assumption is violated, then the Greenhouse-Geisser correction will be used.	As we expect a medium effect size for HR, we will set this effect size as standard for other behavioural hypotheses and will not search for a small effect size.	If p<0.02 on the double interaction term, then the intervention has a different effect on trained “NoGo” than trained “Go” items. In the case that p > 0.02, BF01 should be above 3 to support the absence of interaction.	If HR is confirmed but not H2, we could conclude that the explicit liking is relatively independent of implicit measures of items’ motivational aspect, and thus that a change in explicit liking can happen without a change of implicit wanting. Moreover, it could show that the implicit wanting is not impacted by the intervention.
Will learning bias moderate the intervention’s efficacy?	H3a: A higher sign-tracking bias (higher regression coefficient) will result in a larger reduction in the explicit liking (delta between pre- and post-training) of trained “NoGo” items.	51 participants based on a priori power analysis.<br>The smallest effect size of interest is an R² of .25.<br><br>Will be excluded during analyses:<br>- distribution outliers on the ~learning bias and the explicit liking (above 2.5*MAD around the median).<br>- Error rate above 10% at the Forced Choice task	A linear model with the pre-post-training reduction in explicit liking as the outcome, the learning regression coefficient of CS value on the eye-gaze index as the variable of interest, and the baseline explicit liking as covariate.	We set our SESOI as being an R² of .25 (medium effect) as we are searching for an effect size large enough to be interpretable in our context. A smaller effect will not be considered useful in our approach.	If p<0.02 on the link between the outcome and the variable of interest, then the affective learning bias in question moderates the GNG training effect. In the case that p > 0.02, BF_{01} should be above 3 to support the absence of interaction.	If HR is confirmed but not H3a or H3b, we would conclude that the effect of the bias in question on the intervention’s efficacy, if any, is negligible.
	H3b: A higher model-free bias (lower w) will result in a larger reduction in the explicit liking (delta between pre- and post-training) of trained “NoGo” items.		A linear model with the pre-post-training reduction in explicit liking as the outcome, the weighing parameter w of the model-free/model-based contribution as the variable of interest, and the baseline explicit liking as a covariate.			

Supplementary Table S2. Design table

4. Sample size progression

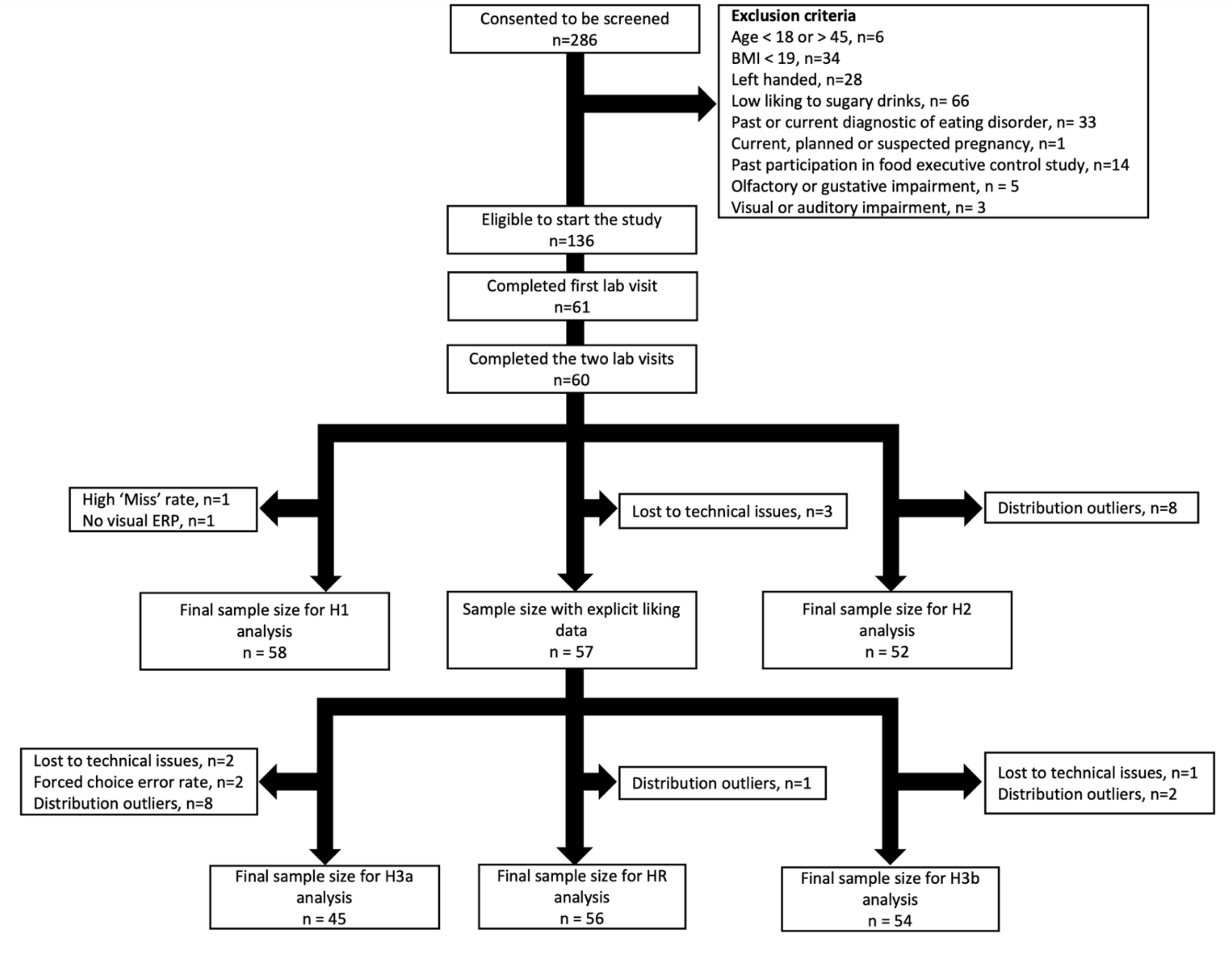

Supplementary Figure S1. Sample size progression

5. Sequential learning computations

5.1 Bias computations

To evaluate the model-free/model-based bias, we will use the reinforcement learning computational modelling as in Daw et al. 2011. and Voon et al. 2015.

The tasks include three stages, first stage being s_A; second stage being s_B or s_C. The action from first stage to second stage is denoted a_A and action between second stage to result (reward or no reward) is denoted a_B.

The objective is to learn the long-run value $Q_{TD}(s, a)$, for each stage s and action a). In a given trial t, the initial state s_A is denoted as $s_{1,t}$, while the subsequent state is noted as $s_{2,t}$. The actions taken in the first and second stages are

referred to as $a_{1,t}$ and $a_{2,t}$, respectively, and the corresponding rewards are identified as $r_{1,t}$ (which is always zero) and $r_{2,t}$ (which is =0.5 CHF or 0 CHF).

Model-free learning

The model-free learning is modelled through the SARSA (λ) temporal differences TD algorithm (Rummery and Niranjan, 1994). During each stage *i* of a given trial *t*, the value associated with the state-action pair that was visited is updated according to:

$$Q_{TD}(s_{i,t}, a_{i,t}) = Q_{TD}(s_{i,t}, a_{i,t}) + \alpha_i \delta_{i,t}$$

where

$$\delta_{i,t} = r_{i,t} + Q_{TD}(s_{i+1,t}, a_{i+1,t}) - Q_{TD}(s_{i,t}, a_{i,t})$$

To update the value associated with the state-action pair in each stage of a given trial, the expressions first incorporate the value of the resulting stage 2 state, $Q_{TD}(s_{2,t}, a_{2,t})$, in order to update the stage-1 action value. Since no reward is received at this stage, the reward $r_{1,t}$ is set to 0. After that, the stage-2 value is updated considering the reward $r_{2,t}$, and the terminal value $Q_{TD}(s_{3,t}, a_{3,t})$, which is defined as 0. Each stage has its own learning rate parameter (α_1, α_2), which is used to update the value associated with that stage. Additionally, the first-stage action value is updated again using the stage-2 prediction error at the end of each trial:

$$Q_{TD}(s_{1,t}, a_{1,t}) = Q_{TD}(s_{1,t}, a_{1,t}) + \alpha_1 \lambda \delta_{2,t}$$

The eligibility trace parameter λ dictates the extent of this update.

Model-based learning

In the model-based reinforcement learning algorithm, the first stage action value (Q_{MB}) takes into account the probability of this action leading to s_B or s_C with $P(s_B|s_A, a_A) = 0.7$ and $P(s_C|s_A, a_A) = 0.3$, or vice versa $P(s_B|s_A, a_A) = 0.3$ and $P(s_C|s_A, a_A) = 0.7$, as well as the values of those states. The second stage (where immediate reward is offered) is modelled through the temporal difference TD algorithm, as the estimated value of action does not require further anticipation. Thus, for each action a_j $(j = A, B)$:

$$Q_{MB}(s_A, a_j) = P(s_B|s_A, a_j) \max_k Q_{TD}(s_B, a_k) + P(s_C|s_A, a_j) \max_k Q_{TD}(s_C, a_k)$$

To connect both values, the net value of action of the first choice is computed as a weighted combination of Q_{TD} and Q_{MB}:

$$Q_{net}(s_A, a_j) = wQ_{MB}(s_A, a_j) + (1 - w)Q_{TD}(s_A, a_j)$$

In which w is the weighting parameter expressing a model-free or model-based biais (w =0 indicates a model-free reliance and w =1 indicates a model-based reliance).
The net value of action of the second choice $Q_{net} = Q_{MB} = Q_{TD}$

Lastly, we employ the softmax equation in Q_{net} to determine the probability of a choice at each stage:

$$P(a_{i,t} = a|s_{i,t}) \propto \exp(\beta_i[Q_{net}(s_{i,t}, a) + p * rep(a)])$$

The primary outcome of this task is thus the weighting parameter w, which will be used to evaluate how the affective learning bias moderates the intervention's efficacy.

Parameters estimation

Parameters were estimated using Maximum A Posteriori (MAP) estimation. Priors were set as follows: beta1 and beta2 were drawn from a gamma distribution, p from a normal distribution, and alpha1, alpha2, w, and lambda were drawn from a logit-transformed normal distribution (Supplementary Table 3).

	**α1**	**α2**	**w**	**β1**	**β2**	**P**	**λ**	**-LP**
mean (n=54)	0.460	0.459	0.475	4.755	3.055	0.132	0.574	219.702
std	0.220	0.297	0.187	2.481	1.054	0.156	0.173	49.678

Supplementary Table S3. Summary of inferred parameters

5.2 Parameters recovery

To assess the reliability of the estimated parameters, we conducted a parameter recovery analysis. We simulated choice behavior for 100 virtual participants, following the task structure described in the Methods section and using the true parameter distributions mentioned above. The simulated data were then fitted using a maximum a posteriori (MAP) estimation approach incorporating prior information. We evaluated recovery by examining the correlation between the original (true) parameters and the recovered estimates. A strong correlation indicates accurate parameter recovery and reliable model estimation. All parameters showed good recovery ($r > 0.55$), and no artificial correlations were introduced between unrelated parameters ($|r| < 0.35$) (Supplementary Figure 2).

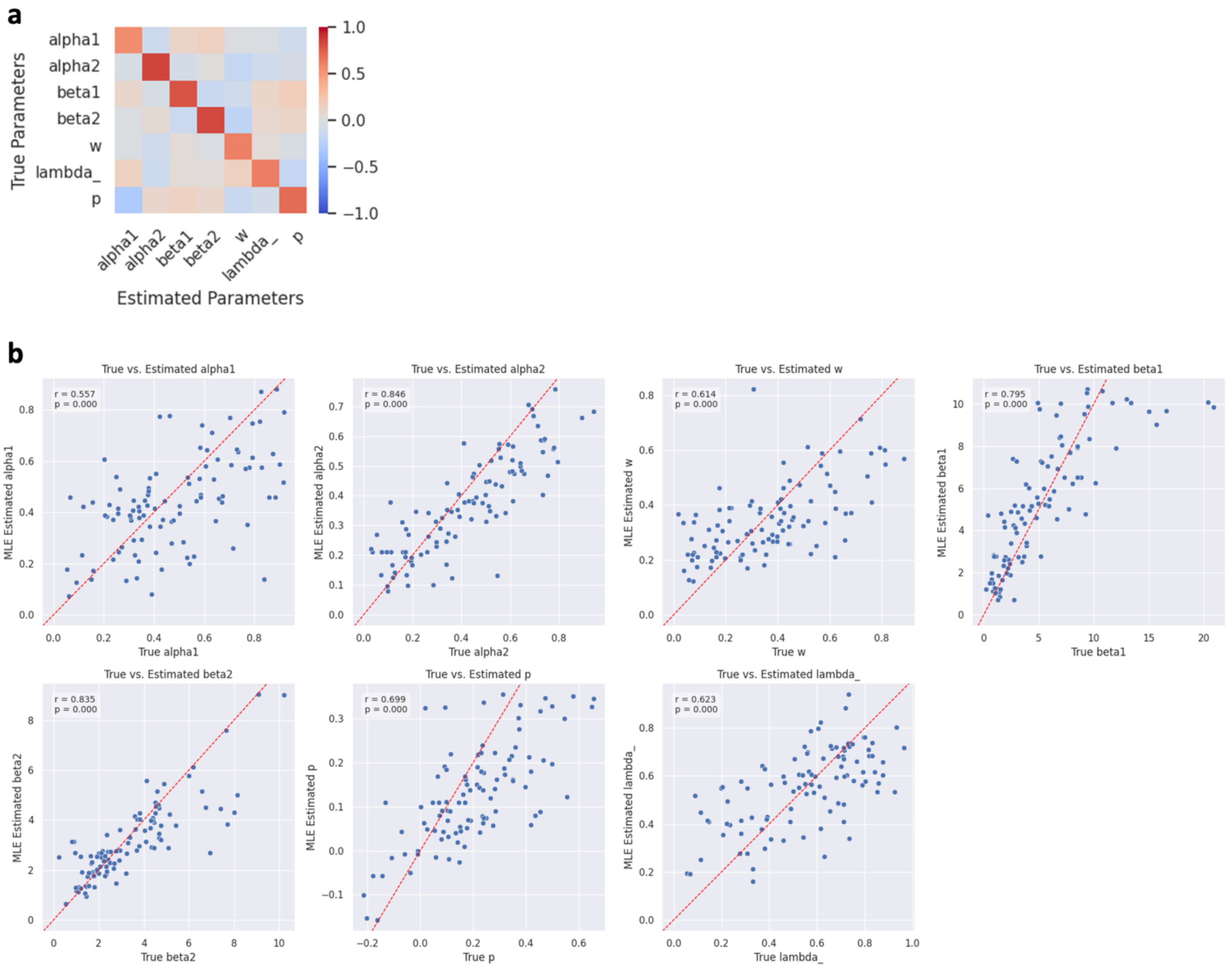

Supplementary Figure S2. Parameter recovery analysis for 100 simulated participants.
(**a**) Correlation matrix showing relationships between true and estimated parameters. All parameters were well recovered (r > 0.55). (**b**) Scatter plots comparing true and estimated values for each parameter, with a strong correspondence between the two, indicating reliable parameter recovery.

6. Pavlovian conditioning analyses

6.1 Regression coefficient computations

To avoid a visual salience effect on the eye gaze at CS presentation, the gaze index is calculated using the last second of CS presentation.

The gaze index is computed as the proportion of fixation time on CS minus the proportion of fixation time on the US location.

Gaze index = p(CS) – p(US)

A linear regression is then performed for each participant on the gaze index with the true value of CS (-2 CHF, -1 CHF, 0 CHF, 1 CHF, 2 CHF) for each participant.

The computed regression coefficient is then used as a moderator of the intervention efficacy. A positive regression coefficient signifies a gaze attracted more to win-predictive than to loss-predictive CSs and thus corresponds to a sign-tracker tendency, whereas a negative coefficient signifies a gaze attracted towards the goal for expected wins more than for expected losses and expresses a goal-tracker tendency.

6.2 Eye-tracking pipeline

The experiment was designed and executed in the software E-prime 3.0 (Psychology Software Tools, Pittsburgh, USA), which commanded a video-based dark-pupil tracking system (SMI iView X RED, version 2.8, Sensomotoric Instruments GmbH, Teltow, Germany) to record eye movement. The system had a temporal resolution of 500 Hz (sampling rate), a spatial resolution of 0.03°, and was able to compensate for head movements. A calibration procedure was performed using the 5-point calibration option. The procedure was run on a screen of 22" in size with a resolution of 1680 x 1050 pixels, 32-bit color depth, and a refresh rate of 60 Hz. Participants were placed at 70cm from the screen. Fractal images had a square dimension of 8.8° and a reward image of 6.5° x 9.56°. The center of the two images had a horizontal distance of 19.5°. Eye-tracking data were analysed using the SMI BeGaze software V.3.7.58 (Sensomotoric Instruments GmbH, Teltow, Germany). Three Areas of Interest (AOIs) were defined for the analysis: a left AOI, a right AOI, and the remaining screen area ("grey space"). The left- and right-defined AOIs corresponded to the squares where the fractals were presented, with an additional 7% margin. Saccades (fixation time < 100ms) and blinks were filtered out, and only fixation events were kept.

Eye-tracking metrics were analysed for the period of interest (i.e., the last second of CS presentation). The tracking accuracy was assessed by computing participants' fixation event tracking performance as the mean of each event's tracking ratio (% of successful eye-tracking during that specific event, expressing the accuracy of the reported event duration). To quantify gaze availability, we computed the proportion of tracked fixation time for each participant by dividing the total recorded fixation time by the total duration of the analysed window (80 trials × 1 s). This measure therefore reflects the proportion of the analysis period for which valid fixation data were recorded.

Our analysis indicates adequate eye-tracking performance: event's tracking accuracy was high (mean tracking performance across participants = 95.3% ± 4.5), indicating adequate eye-tracking performance. The proportion of recorded fixation time was also high (mean recorded fixation time across participants = 72.4% ±16.8), indicating that only around 27.6% of the recorded window did not contain valid fixation samples (e.g., due to blinks, saccades, or lost signal)

AOIs' fixation proportion were computed for each participant by dividing the total fixation time within each AOI by the total recorded fixation time. Fixations were predominantly directed towards the left (48.8% ± 8.6) and right (45.1% ± 7.8) ROIs, with relatively little time spent fixating on the grey space (6.4% ± 11.9). This indicates that gaze was mainly concentrated on the regions of interest, consistent with expected task engagement.

7. Order effect analysis on learning biases

To evaluate whether the learning bias (i.e., pavlovian bias or w) differed when they were administered pre- or post-training. We conducted an analysis to predict learning bias based on visit order.

For the Pavlovian learning bias, the bias estimates did not differ across task timing orders. A linear regression predicting bias (beta1) from visit showed no evidence of an order effect, $\beta = 0.0014$, SE = 0.0101, $t(43) = 0.14$, $p = .890$. The model explained almost no variance in bias ($R^2 = 0.001$).

For the bias w, a linear regression predicting the outcome from visit showed no evidence of a timing/order effect, $\beta = -0.055$, SE = 0.049, $t(48) = -1.13$, $p = .263$. The model explained little variance ($R^2 = 0.026$).

8. Electrical Source Estimations

Brain sources of ERP modulations were estimated using a distributed linear inverse solution model (a minimum norm inverse solution) combined with the local autoregressive average (LAURA) regularization approach, which describes the spatial gradient across neighboring solution [2,3]. LAURA enables investigating multiple simultaneously active sources and selects the configuration of active brain networks that better mimics biophysical behavior of neural

fields. LAURA uses a realistic head model, and the solution space included 3005 nodes, selected from a grid equally distributed within the gray matter of the Montreal Neurological Institute's average brain. The head model and lead field matrix were generated with the spherical model with anatomical constraints (SMAC; [4]). As an output, LAURA provides current density measures; their scalar values were evaluated at each node. Assessments of the localization accuracy of this inverse solution by fundamental and clinical research indicate that the estimations and the results of their statistical analyses can be confidently interpreted at the resolution of the grid size (here 6 mm; e.g., [3,5–7]). To correct for multiple testing and spatial autocorrelation, we will apply a spatial-extent correction (Ke) of ≥15 contiguous nodes with a P-value < 0.05.

9. Go/NoGo task

9.1 EEG analysis

EEG pre-processing results

n = 58	Mean ±SD
Number of interpolated electrodes	9.60 ±5.71 (15%)
Percentage of epochs with 80uV artefacts	1.12 ±4.07
Percentage of epochs with 30uV jumps	0 ±0
Number of kept epochs	146.38 ±9.63

Supplementary Table S4. EEG pre-processing results

Segmentation interval

P1 (ms)	Onset	Offset	GFP Peak	Length
Pre-training Trained Go	65	106	85	41
Post-training Trained Go	67	104	86	37
Pre-training NoGo	65	106	85	41
Post-training NoGo	66	105	85	39
After GFP-locking, across all conditions	19ms before GFP peak	18ms after GFP peak	-	38

Supplementary Table S5. P1 segmentation intervals

N2 (ms)	Onset	Offset	GFP Peak	Length
Pre-training Trained Go	172	271	220	99
Post-training Trained Go	171	272	224	101
Pre-training NoGo	181	271	220	90
Post-training NoGo	172	266	224	94

After GFP-locking, across all conditions	39 ms before GFP peak	42 ms after GFP peak	-	82

Supplementary Table S6. N2 segmentation intervals

<u>Trained Go control</u>

For P1, there were 5 consecutive significant TFs starting at the onset of P1 for the GFP, and 2 consecutive significant TFs starting at 36ms for the GMD, resulting in no significant results for this component (see Supplementary Figure 3).

For N2, there were 84 consecutive significant, spanning the whole component, for both the GFP and GMD, resulting in a significant effect of training on the Trained Go items for this component (see Supplementary Figure 3).

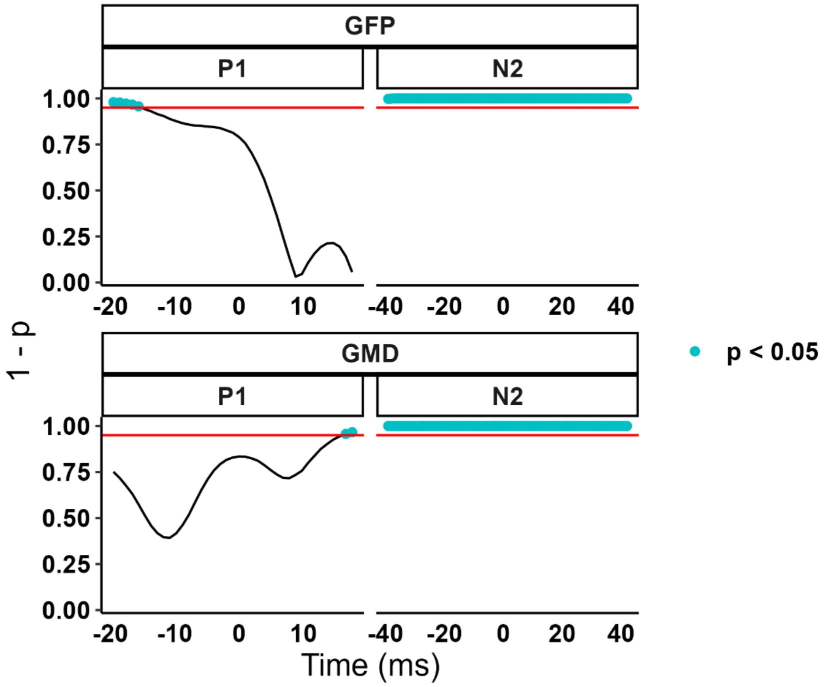

Supplementary Figure S3. Inverted p-values (y-axes; blue dot when significant) are represented at each ms (x-axes) for both P1 and N2 ERP components (left and right column) and for both the GFP and GMD analyses (first and second rows). The alpha threshold (0.05) is represented with a red horizontal line.

10. Results tables

Results tables for each tested hypotheses are reported below.

10.1 HR) Training-induced modifications of explicit liking of the "NoGo" trained cues

Mean ±SD	Trained as "Go"		Trained as "NoGo"		
	**Pre**	**Post**	**Pre**	**Post**	**Training condition x session**
**Explicit liking (%)**	**73.7** ±13.0	**67.2** ±12.0	**73.8**±13.1	**63.4** ±13.5	$F_{[1,55]}$ = 8.41<br>p = 0.005<br>BF_{01} = 0.81

**Pre-Post delta [95% CI]**	6.52 [3.65 ; 9.39]	10.31 [7.40 ; 13.22]

Supplementary Table S7. Explicit liking results

10.2 H2) Training-induced modifications of implicit wanting of the "NoGo" trained cues

**Mean** ±SD	**Trained as "Go"**		**Trained as "NoGo"**		
	**Pre**	**Post**	**Pre**	**Post**	**Training condition x session**
**Implicit Wanting (RT.away–RT.towards) (s)**	0.042 ± 0.058	0.048 ± 0.044	0.044 ± 0.057	0.053 ± 0.048	$F_{[1,51]}$ = 0.013<br>p = .910<br>BF_{01} = 4.86
**Pre-Post delta [95% CI]**	-0.01 [-0.03; -0.01]		-0.01 [-0.03; 0.01]		

Supplementary Table S8. Implicit wanting results

10.3 H3) Moderating effect of learning biases on the reduction in "NoGo" cues explicit liking

**Predictor**	**Estimate (β) ± SE**	**t(df)**	**p-value**	**BF_{01}**	**R^2**	**DF**
			Sign-tracking bias			
**Intercept**	-1.40 ± 7.54	-0.186	0.854	-	0.207	42
**Regression coefficient**	107.94 ± 38.70	2.79	0.008	0.141	-	-
**Baseline explicit liking**	0.14 ± 0.10	1.4	0.169	-	-	-
			Model-free bias			
**Intercept**	-1.51 ± 7.44	-0.203	0.840	-	0.097	51
**w**	-7.20 ± 6.84	-1.053	0.297	1.828	-	
**Baseline explicit liking**	0.215 ± 0.096	2.229	0.030	-	-	-

Supplementary Table 9. Affective learning bias results

11. Implicit wanting exploratory analysis

11.1 Session effect on reaction times

RT towards

Twelve distribution outliers were excluded, the final analysis thus included 47 participants. A summary of the results can be found in the Supplementary Figure 4.

For the Condition x Session interaction on reaction times towards sugary drinks, the homoscedasticity assumption was respected (Levene's Test: $F(3, 184) = 0.20$, $p = .895$). There was a significant effect of session (ANOVA: $F(1,46) = 63.68$, $p < .005$, partial $\eta^2 = .58$). The double interaction term (Session x Condition) was not significant (ANOVA: $F(1,46) = 0.001$, $p = .97$, partial $\eta^2 = .00$)

RT away

Thirteen distribution outliers were excluded, the final analysis thus included 47 participants. A summary of the results can be found in the Supplementary Figure 5.

For the Condition x Session interaction on reaction times away from sugary drinks, the homoscedasticity assumption was respected (Levene's Test: $F(3, 180) = 0.39$, $p = .760$). There was a significant effect of session (ANOVA: $F(1,45) = 60.29$, $p < .005$, partial $\eta^2 = .57$). The double interaction term (Session x Condition) was not significant (ANOVA: $F(1,46) = 0.117$, $p =.73$, partial $\eta^2 = .00$)

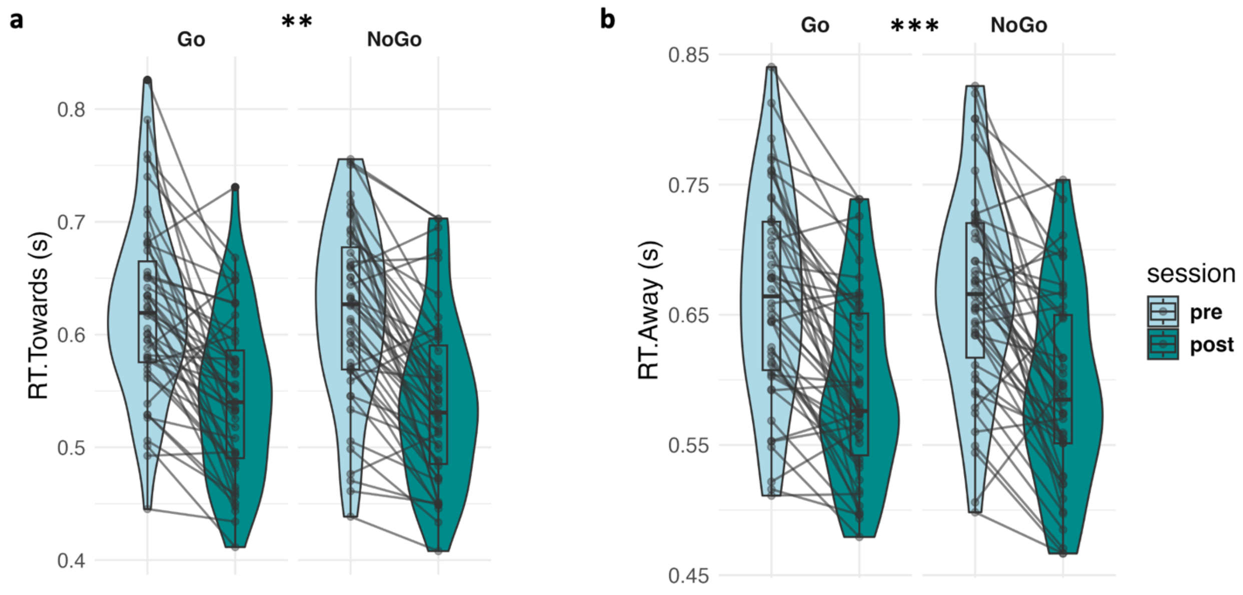

Supplementary Figure S4. Reaction times during the SRC task. a) RT towards sugary drinks at pre- and post-intervention for items trained as "Go" and "NoGo". b) RT away sugary drinks at pre- and post-intervention for items trained as "Go" and "NoGo".

11.2 Task reliability

A permutation-based split-half reliability analysis was conducted using the rapidsplithalf R package[8].

The split-half reliability of the IW was *r*SB(50) = 0.48, 95% CI [0.16, 0.7] (6000 permutations; Spearman-Brown coefficient; Supplementary Figure 4), indicating that IW estimates contain substantial measurement noise.

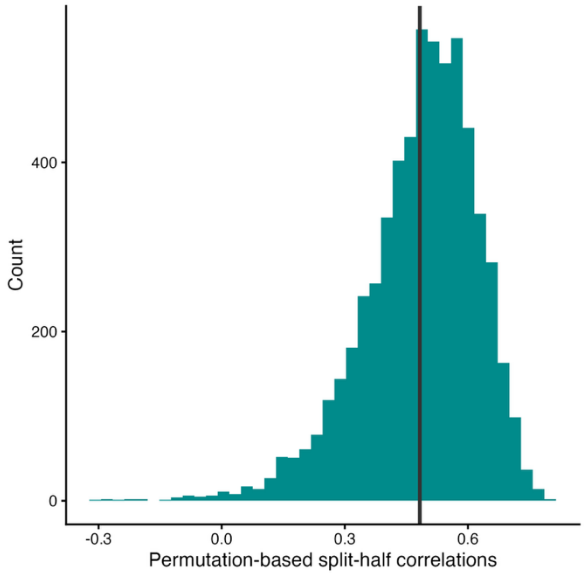

Supplementary Figure S5. Histogram of permutation-based split-half correlations (6000 random splits) for the IW score. The vertical line indicates the Spearman-Brown corrected full-length reliability estimate.

12. Bibliography

1. Tzavella, L. & Chambers, C. D. Explicit and implicit devaluation effects of food-specific response inhibition training. *J. Cogn.* **6**, (2023).
2. Grave De Peralta Menendez, R., Murray, M. M., Michel, C. M., Martuzzi, R. & Gonzalez Andino, S. L. Electrical neuroimaging based on biophysical constraints. *NeuroImage* **21**, 527–539 (2004).
3. Menendez, R. G. D. P., Andino, S. G., Lantz, G., Michel, C. M. & Landis, T. Noninvasive localization of electromagnetic epileptic activity. I. Method descriptions and simulations. *Brain Topogr.* **14**, 131–137 (2001).
4. Spinelli, L., Andino, S. G., Lantz, G., Seeck, M. & Michel, C. M. Electromagnetic inverse solutions in anatomically constrained spherical head models. *Brain Topogr.* **13**, 115–125 (2000).
5. Gonzalez Andino, S. L., Michel, C. M., Thut, G., Landis, T. & Grave de Peralta, R. Prediction of response speed by anticipatory high-frequency (gamma band) oscillations in the human brain. *Hum. Brain Mapp.* **24**, 50–58 (2005).
6. Gonzalez Andino, S. L., Murray, M. M., Foxe, J. J. & Menendez, R. G. D. P. How single-trial electrical neuroimaging contributes to multisensory research. in *Experimental Brain Research* vol. 166 298–304 (Springer, 2005).
7. Michel, C. M. *et al.* EEG source imaging. *Clin. Neurophysiol.* **115**, 2195–2222 (2004).
8. Kahveci, S., Bathke, A. C. & Blechert, J. Reaction-time task reliability is more accurately computed with permutation-based split-half correlations than with Cronbach's alpha. *Psychon. Bull. Rev.* **32**, 652–673 (2025).
